# Making Sense of Misfortune: Deservingness, Self-Esteem, and Patterns of Self-Defeat

**DOI:** 10.1037/a0036640

**Published:** 2014-07

**Authors:** Mitchell J. Callan, Aaron C. Kay, Rael J. Dawtry

**Affiliations:** 1 Department of Psychology, University of Essex; 2 The Fuqua School of Business, Duke University; 3 School of Psychology, University of Kent

**Keywords:** deservingness, self-esteem, just-world theory, self-defeating, self-punishment

## Abstract

Drawing on theorizing and research suggesting that people are motivated to view their world as an orderly and predictable place in which people get what they deserve, the authors proposed that (a) random and uncontrollable bad outcomes will lower self-esteem and (b) this, in turn, will lead to the adoption of self-defeating beliefs and behaviors. Four experiments demonstrated that participants who experienced or recalled bad (vs. good) breaks devalued their self-esteem (Studies 1a and 1b), and that decrements in self-esteem (whether arrived at through misfortune or failure experience) increase beliefs about deserving bad outcomes (Studies 1a, 1b, 2a, 2b). Five studies (Studies 3–7) extended these findings by showing that this, in turn, can engender a wide array of self-defeating beliefs and behaviors, including claimed self-handicapping ahead of an ability test (Study 3), the preference for others to view the self less favorably (Studies 4–5), chronic self-handicapping and thoughts of physical self-harm (Study 6), and choosing to receive negative feedback during an ability test (Study 7). The current findings highlight the important role that concerns about deservingness play in the link between lower self-esteem and patterns of self-defeating beliefs and behaviors. The theoretical and practical implications of these findings are discussed.

Most people can remember an occasion when through bad luck or bad timing they experienced a negative outcome, such as ending up on the losing side of a coin flip, failing an important task despite their best intentions and efforts, or accidently causing someone else harm or embarrassment. Anecdotal observation and experimental evidence suggest that such negative experiences, despite being unintended and unforeseeable, often elicit feelings of guilt and sadness (e.g., Freedman, Wallington, & Bless, 1967; McGraw, 1987; Meindl & Lerner, 1984; Miller & Gunasegaram, 1990). Perhaps even more intriguing and puzzling is research showing that people may be so moved by these experiences that they will even devalue themselves (e.g., Apsler & Friedman, 1975; Rubin & Peplau, 1973), selectively remember their personal shortcomings (Callan, Kay, Davidenko, & Ellard, 2009), or cause harm to themselves (e.g., Comer & Laird, 1975; Ferrari, 1990; Walster, Aronson, & Brown, 1966; for a review, see Baumeister & Scher, 1988). For instance, Comer and Laird (1975) found that a large majority of their participants who were randomly assigned to suffer an ill-fate (i.e., to eat a live worm) later *chose* to suffer the same ill-fate even when they were given the opportunity to opt for a less aversive outcome. In a similar vein, innocent victims of extreme injustices (e.g., rape victims) sometimes try to breathe meaning into their experiences by devaluing, or somehow finding fault in, themselves (Davis, Lehman, Silver, Wortman, & Ellard, 1996; Hall, French, & Marteau, 2003; Janoff-Bulman, 1992; Littleton, Magee, & Axsom, 2007; Miller & Porter, 1983).

Such self-defeating beliefs and behaviors following experiences of random negative outcomes are as surprising as they are seemingly irrational. Indeed, societal conventions governing the assignment of responsibility and blame generally absolve anyone of culpability when negative outcomes are unintended, unforeseeable, or uncontrollable (see Shaver, 1985; Weiner, 1995). Thus, by commonly accepted standards, people ought not to condemn themselves for negative outcomes brought about by chance or factors beyond their control. Moreover, these self-defeating beliefs and behaviors are seemingly at odds with the prevailing view that humans are fundamentally motivated to both maximize pleasure and minimize pain (Schwartz, 1986; Wilson, 1978) and generally view themselves in as positive a light as possible (Leary, 2007; Sedikides & Gregg, 2008). Why, then, would people be willing to devalue or punish themselves for outcomes that they, by all reasonable accounts, did not cause?

The notion that people may, at times, adopt self-defeating beliefs and behaviors following negative experiences may become less of a paradox in light of what we know about the psychology of deservingness (Feather, 1999; Hafer, 2011; Jost & Kay, 2010; Lerner, Miller, & Holmes, 1976; Major, 1994). Specifically, theorizing and research—much of it originating with Lerner’s (1980) Just World Theory—indicates that people need to maintain the belief that the world is basically an orderly, non-random place where people—including ourselves—get what they deserve and deserve what they get (Hafer & Bègue, 2005; Lerner, 1980). Because maintaining deservingness beliefs serves an adaptive function (Callan, Harvey, Dawtry, & Sutton, 2013; Hafer, 2000; Laurin, Fitzsimons, & Kay, 2011; Lerner, 1977), people often rationalize their experiences, even unjust ones, in order to maintain perceptions of deservingness (for reviews, see Callan & Ellard, 2010; Hafer & Bègue, 2005; Jost & Kay, 2010; Lerner, 1980). That is, they often go to great lengths to maintain their beliefs that rewards and punishments are not haphazard but are doled out according to an understandable set of rules (Kay, Gaucher, Napier, Callan, & Laurin, 2008). Might this theoretical perspective—which emphasizes a powerful human motive to view outcomes as deserved rather than random—contribute to our understanding of self-defeating beliefs and behaviors?

To answer this question, we leveraged classic and contemporary theory and research to shed light on one potential mechanism underlying self-defeating beliefs and behaviors among “normal,” nonclinical samples. We adopted Baumeister and Scher’s (1988) definition of self-defeating behavior as “any deliberate or intentional behavior that has clear, definitely or probably negative effects on the self or the self’s projects” (p. 3). Specifically, we tested the general idea that, to the extent people are motivated to view the world as an orderly place that conforms to rules of deservingness, they might adopt various self-defeating beliefs and behaviors (e.g., self-handicapping, thoughts of self-harm, choosing to self-punish) following the experience of misfortune because people feel compelled to view misfortune—even though, by definition, it is uncontrollable—as *deserved.*

In one of the earliest treatments of Just World Theory, Lerner et al. (1976) defined “deserving” as a judgment that follows the perception of an appropriate relation between the value of people or their actions and the value of their outcomes (see also Feather, 1999). By this characterization, bad outcomes, for example, are deemed deserved by virtue of who the recipients are (bad people) or what they have done (bad deeds). Research has confirmed that observers judge bad (good) people as deserving of their fortuitous bad (good) fortunes (e.g., Callan, Sutton, Harvey, & Dawtry, 2014; Pepitone & L’Armand, 1996; Rice & Trafimow, 2011), and the innocent suffering of devalued (vs. valued) individuals and groups as less unfair (e.g., Callan, Dawtry, & Olson, 2012; Callan, Powell, & Ellard, 2007; Correia, Vala, & Aguiar, 2007; Lerner & Agar, 1972). Crucially, people also *expect* bad (good) things to happen to bad (good) people (e.g., Callan, Ferguson, & Bindemann, 2013; Kaplowitz, 1979). For example, Callan, Ellard, and Nicol (2006) found that participants rated a man who cheated on his wife as more deserving of a completely unrelated car accident than participants who learned the man did not cheat. Further, early experimental evidence revealed that observers can satisfy their motive to view outcomes as driven by deservingness by inferring others’ moral worth simply on the basis of their fortuitously experienced good or bad *outcomes* (e.g., Apsler & Friedman, 1975; Lerner, 1965; Lerner & Simmons, 1966; see Lerner & Miller, 1978). That is, people appear fundamentally motivated to view that bad (good) things happen to bad (good) people.

These findings highlight the role that a concern for deservingness plays in people’s reactions to the fates of others. Might the same processes operate in the context of considering one’s own outcomes and self-worth? That is, might people over-apply models of deservingness to explain their own seemingly chance fortunes? If so, we would expect people to literally feel worse about their self-worth—that is, evidence lower self-esteem—after experiencing misfortune. Consistent with this, research has shown that people devalue (or enhance) their attributes and self-esteem when chance negative (or positive) outcomes occur (e.g., Apsler & Friedman, 1975; Callan, Kay, et al., 2009; Dion & Dion, 1987; Ellard & Bates, 1990; Janoff-Bulman, 1992; Rubin & Peplau, 1973). For example, in a natural experiment, Rubin and Peplau (1973) found that young men who learned through a random draft lottery that they would likely be drafted to serve in Vietnam significantly lowered their self-esteem compared to men who learned they would likely not be drafted. As Lerner argued (1998; see also Lerner & Clayton, 2011), such experiences of good or bad fortunes, however fortuitous they might be, can lead to corresponding changes in self-evaluations, due to the application of “causal schemas or scripts that initially appeared very early in [people’s] lives and persist throughout adulthood—bad outcomes are caused by bad people” (Lerner, 1998, p. 255). That is, the motive to believe that people get what they deserve, Lerner theorized, can lead people to justify their current negative experiences by devaluing, or finding fault, in themselves.

Theoretically, though, this psychological chain may not end here. That is, to the extent people are in fact motivated to view the world this way, not only should their views of self-worth be affected by the misfortunes that befall them, but their feelings of self-worth should affect what they feel they deserve. Indeed, a handful of studies suggest that people lower in self-esteem feel more deserving of bad outcomes (Feather, 2006; Heuer, Blumenthal, Douglas, & Weinblatt, 1999; Wood, Heimpel, Manwell, & Whittington, 2009). For example, Wood et al. (2009) found that participants who were either situationally or chronically low in self-esteem felt that they did not deserve to experience positive emotions. However, although *deservingness* has long been invoked as a mechanism to explain people’s responses to their own good or bad outcomes, it has rarely been measured directly. In Wood et al.’s words, “such studies have not measured participants’ sense of deservingness, so we cannot be sure that deservingness was important” (p. 364). Moreover, little research has been focused on the variety of potential self-defeating beliefs and behaviors than can arise from people’s beliefs that they deserve bad outcomes in life, and none has focused on this collection of processes in the context of outcomes that individuals have little control over.

Integrating these various theoretical approaches, we test the general hypotheses that self-esteem (a) will be impacted by the experience of random (mis)fortune, (b) will lead to corresponding changes in feelings of deservingness, and (c) will lead people to adopt self-defeating beliefs and engage in self-defeating behaviors. What is more, across 7 studies that adopt various experimental and correlational designs, we illustrate the feasibility of a causal chain that begins with the experience of random (mis)fortune and, by way of corresponding changes in self-esteem and feelings of deservingness, ends in the adoption of self-defeating beliefs and behaviors.

The conceptual framework for our studies is outlined in Figure 1. The variables we manipulated or measured for each of the 7 studies are shown with solid lines under the figure. By way of summary, in Studies 1a and 1b, we examine the effects of experiencing/recalling breaks on state self-esteem and beliefs about deserving bad outcomes. In Studies 2a and 2b, we manipulate state self-esteem to determine the causal role of state self-esteem in producing changes in beliefs about deserving bad outcomes. In Studies 3a and 3b, we examined the effects of experiencing/recalling bad breaks on self-handicapping. In Study 4, we examined the effect of recalling bad breaks on preferred appraisals by close others. In Studies 5–7, we examined the mediating role of beliefs about deserving bad outcomes in the link between trait self-esteem and preferred appraisals by others (Study 5), chronic self-handicapping (Study 6), thoughts of self-harm (Study 6), and choosing to self-punish (Study 7). Across of all these studies, then, we aimed to demonstrate (a) that people’s need to view their world as just and predictable—that is, as one in which outcomes, good and bad, are distributed to those who deserve them—can engender a wide array of self-defeating beliefs and behaviors; (b) that self-esteem plays a key role in this process; and (c) that because of a concern for deservingness, even random and mundane misfortunes are sufficient to trigger this chain of events.[Fig-anchor fig1]

## Studies 1A and 1B: Effects of Good/Bad Breaks on Self-Esteem and Deservingness of Bad Outcomes

We first examined the effects of experiencing or recalling random bad breaks on self-esteem and corresponding beliefs about deserving bad outcomes. To the extent that people are motivated to believe the world is an orderly place where people get what they deserve, experiencing/recalling bad (vs. good) breaks should lead people to devalue their state self-esteem, because self-esteem may be an indicator of how good versus bad breaks are being internalized and perceived as reflecting directly on the self. Past research suggests this should be the case (see Lerner, 1998). What is not known, however, is the consequences of these changes in state self-esteem for people’s subsequent perceptions of what they deserve in their immediate future. In these two studies, we aimed to establish empirically that reductions in positive state self-esteem arising from the experience of random bad breaks translate into increased beliefs about deserving bad outcomes in the future. If people are motivated to believe that bad (good) people deserve bad (good) outcomes (Lerner, 1980), then changes in self-esteem should lead to concomitant changes in beliefs about deserving bad outcomes. The belief that one *deserves* bad outcomes, by definition (Lerner et al., 1976), requires a less favorable view of the self—bad people deserve bad outcomes. Therefore, we propose that self-evaluations causally precede specific deservingness beliefs that, in turn, affect self-defeating beliefs and behaviors.

After either experiencing a bad (vs. good) break (Study 1a) or recalling recent bad (vs. good) breaks (Study 1b), participants completed a state self-esteem scale and a scale designed to measure the extent to which they believed they deserved bad outcomes. We hypothesized that the effect of experiencing/recalling bad breaks on increased beliefs about deserving bad outcomes would be mediated by changes in state self-esteem. One possible alternative account of this prediction is that instead of specific changes in self-evaluations, recalling/experiencing bad versus good breaks lead to changes in affect that, in turn, “infuse” into judgments of deserving bad outcomes (e.g., Forgas, 1995). Thus, we also measured positive and negative affect to ascertain the specific mediating role of state self-esteem in the effect of experiencing/recalling bad versus good breaks on beliefs about deserving bad outcomes, conditional on the potential mediating role of general affects.

### Method

#### Study 1a

##### Participants

We approached 76 people at various locations around the University of Essex, United Kingdom, campus to complete a “personality study.” Data from two participants were not included in analyses because they inadvertently completed the manipulation procedure *after* the dependent measures. The resulting sample composed 30 males and 43 females (1 unreported; *M*_age_ = 25.22 years, *SD*_age_ = 11.26). Participants were given a small candy bar and £3 for their participation.

##### Materials and procedure

After initially agreeing to take part in the study for a small candy bar, participants were presented a questionnaire package, the cover sheet of which contained a consent form and, underneath, an introduction to a “Peel n’ Reveal Lottery” (PnR). Participants learned that as an additional incentive for their participation, we were giving them the opportunity to win or “lose” £3. Beneath the instructions was a rectangular box covered by a plain white sticky label (25 mm × 50 mm), mounted over clear tape to prevent tearing, with a colored pull tab protruding from the right hand side. Participants were told to peel off the tab to discover if they won or lost. Peeling off the label revealed text underneath reading either WIN or LOSE. This served as our manipulation of a random good or bad break. Blind to the experimenter, WIN and LOSE questionnaires were randomly distributed to participants (all participants were eventually paid £3 regardless of the outcome of the lottery).

On the following page participants were asked: “For our records, please indicate whether you won or lost the Peel n’ Reveal Lottery,” and were required to circle the appropriate response (WON or LOST). Immediately after affirming the good or bad break, participants completed a state version of Rosenberg’s (1965) Self-Esteem Scale (SES; e.g., “Right now, I feel that I am a person of worth, at least on an equal basis with others”; α = .85). Embedded within the SES were three items designed to measure participants’ beliefs about deserving bad outcomes: “Right now, I believe I am deserving of all the good things life has to offer” (reverse-scored); “Right now, I do not feel deserving of positive outcomes”; and “Right now, I feel I deserve good luck.”^1^ Participants responded to all items using a scale ranging from 1 (*strongly disagree*) to 6 (*strongly agree*).

Participants also completed 7 positive (e.g., *excited, happy, satisfied, cheerful, encouraged, joyous, relaxed*; α = .88) and 9 negative (e.g., *distressed, upset, afraid, guilty, scared, angry, ashamed, irritable, sad*; α = .85) items from the Positive and Negative Affect Schedule (PANAS; Watson, Clark, & Tellegen, 1988). Participants rated the extent to which they were presently experiencing each of the 16 emotions on a 6-point scale (1 = *not at all*, 6 = *very much*).

#### Study 1b

##### Participants

We recruited participants online (*N* = 218, 45% male; *M*_age_ = 31.28 years, *SD*_age_ = 11.92) using Amazon’s Mechanical Turk (MTurk; Buhrmester, Kwang, & Gosling, 2011).

##### Materials and procedure

Participants learned that the study concerned “aspects of personality and daily experiences.” They first completed a survey of a “content analysis of life experiences” where they were asked to recall either 4 good breaks or 4 bad breaks (cf. Gaucher, Hafer, Kay, & Davidenko, 2010). Participants read:

For this part of the survey, we’re interested in people’s life experiences. Specifically, we’re currently interested in learning about the kinds of good [bad] breaks people experience in their lives. “Good breaks” [“Bad breaks”] are those sorts of positive [negative] experiences we have that we do not intend, expect, or plan to occur—they just happen to us. Please list below 4 good [bad] breaks that you have experienced in your life.

Next, participants completed a state version of Rosenberg’s SES (α = .94), a six-item Deservingness of Bad Outcomes Scale (DBOS; see Table 1), and the PANAS items from Study 1 (αs > .91). Shown in Table 1, the items for the DBOS were adapted and extended from scales constructed by Wood et al. (2009). The items were written to reflect participants’ beliefs about deserving bad and good outcomes (with the items framed in terms of good outcomes reverse scored). All six items from the DBOS loaded onto a single principal component (eigenvalue = 4.03, 67.12% of the variance accounted for) and the scale demonstrated acceptable internal consistency (α = .91).[Table-anchor tbl1]

### Results

Shown in Table 2, independent samples *t*-tests showed that participants who experienced a bad (vs. good) break during the PnR (Study 1a) significantly viewed themselves more negatively and believed they were more deserving of bad outcomes (the good and bad break conditions were coded as 0 and 1, respectively, for both Studies 1a and 2b). One participant from the bad breaks condition reported exceptionally low self-esteem (studentized deleted residual = 4.64; Cook’s *D* that was 7.54 *SD* above the mean of Cook’s *D* in these analyses) and was not included in this analysis. Including this datum results in a stronger effect of breaks on self-esteem, *t*(72) = 2.57, *p* = .01, *d* = 0.61.[Table-anchor tbl2]

Similarly, in Study 1b, independent samples *t*-tests showed that participants who recalled bad breaks had lower state self-esteem and more strongly believed they deserved bad outcomes compared to participants who recalled good breaks. The effect of experienced/recalled breaks on positive and negative affect was mixed across Studies 1a and 1b, with participants feeling significantly more negative affect when they recalled bad (vs. good) breaks in Study 1b.

Separately for Studies 1a and 1b, we used Preacher and Hayes’s (2008) multiple mediation bootstrapping procedure (10,000 resamples) to test the indirect effects of the good/bad breaks manipulations on deserving bad outcomes through self-esteem, negative affect, and positive affect. Analysis revealed bias-corrected and accelerated 95% confidence intervals (95% BCa CI) of .05 and .50 (total effect = .30; indirect effect = .27, *SE* = .11) and .15 to .64 (total effect = .40; indirect effect = .38, *SE* = .13) for self-esteem for Studies 1a and 1b, respectively. Neither negative affect nor positive affect significantly mediated the effect of experiencing/recalling bad breaks on deserving bad outcomes for either study (indirect effects ranged from –.01 to 04, all confidence intervals crossed zero). These analyses suggest that, as predicted, lower state self-esteem mediates the effect of experiencing/recalling bad (vs. good) breaks on increased beliefs about deserving bad outcomes. A similar pattern was not observed for positive and negative affect, suggesting that general affect does not mediate the effect of experiencing/recalling bad breaks on beliefs about deserving bad outcomes in the same way as state self-esteem.

## Studies 2A and 2B: Self-Esteem and Deservingness of Outcomes

Studies 1a and 1b revealed that experiencing/recalling bad (vs. good) breaks reduced participants’ state self-esteem which, in turn, increased their beliefs about deserving subsequent bad outcomes. Although this mediation pattern conformed to our predictions, the causal direction between self-esteem and beliefs about deserving bad outcomes could not be determined because both constructs were assessed simultaneously. Our theoretical perspective suggests that, by definition, to believe that one deserves bad outcomes requires a less favorable view of the self (Lerner et al., 1976). In Studies 2a and 2b, then, we tested this process directly by adopting an experimental-causal-chain approach to testing psychological process, which involves manipulating the proposed mediator (self-esteem) to show changes in the focal outcome variable (deservingness; Spencer, Zanna, & Fong, 2005). In Study 2a, we manipulated self-esteem by informing participants that they “failed” or “succeeded” an intelligence test via a false feedback manipulation (e.g., Cameron, Holmes, & Vorauer, 2009; Dodgson & Wood, 1998; Hayes, Schimel, & Williams, 2008). Participants then rated the fairness and reasonableness of a subsequent random good or bad break. We expected that participants who “succeeded” in the test would rate the random outcome as more fair when they experienced a good (vs. bad) break. This “positivity bias” (Mezulis, Abramson, Hyde, & Hankin, 2004; Taylor & Brown, 1988), however, should be significantly dampened when participants learned they “failed” the test. Put differently, given our theoretical perspective, participants who “failed” the intelligence test should perceive the good (bad) break as less (more) fair than participants who “succeed” the test.

If people are motivated to maintain an appropriate relation between their personal worth and the value of their (even random) outcomes, then participants situationally low (high) in self-esteem should judge a random bad (good) break as more fair and reasonable. In Study 2b, participants listed the attributes they least liked about themselves (vs. control; Wood et al., 2009) and then rated the extent to which they believed they deserved bad outcomes in life. We predicted that participants who thought about and listed their least favorable qualities and attributes (vs. participants who thought about “neutral” aspects of their lives) would believe they were more deserving of bad outcomes.

### Method

#### Study 2a

##### Participants

Ninety students from the University of Essex participated in a laboratory experiment for £4 (plus £3, see below). Seven participants were not included in analyses because they did not complete the PnR procedure at the correct time or were suspicious of the feedback they received about their non-verbal reasoning ability. The resulting sample consisted of 83 participants (43.4% male, 1.2% unreported; *M*_age_ = 25.79 years, *SD*_age_ = 5.31).

##### Materials and procedure

###### Feedback manipulation

Participants learned that the study concerned assessing non-verbal reasoning. They first completed what was ostensibly a computer-based non-verbal reasoning (NVR) test, which was in fact an elaborate Microsoft PowerPoint presentation and formed our success or failure feedback manipulation. Participants were first presented with an instruction screen describing that they were required to solve Raven’s matrices under a time constraint of 30 s per matrix. Participants were informed that response times were used in calculating their score, and that missing responses would be treated as incorrect. These instructions were intended to encourage participants to respond quickly and to make a response on every trial, which in turn was expected to make it more difficult for participants to accurately judge their absolute level of performance on the task. Participants were also informed that, “responses will be automatically cross-referenced with population data at the end of the task in order to calculate your standardized NVR score, and you will receive feedback on your performance.”

Participants then completed a series of 24 matrices, presented one per slide, selected from Raven’s (1938) Standard Progressive Matrices, eight of which were judged to be relatively easy, eight intermediate, and eight difficult. Matrices were presented in order of increasing difficulty. On each trial participants were required to identify the correct missing piece and respond by clicking the corresponding number on an on-screen number pad underneath the matrix set. The number pad consisted of animated buttons labeled 1–8. Clicking any button caused the presentation to advance to the next slide, or the presentation would automatically advance after 30 s. A decreasing timer bar at the bottom of the screen showed the remaining time available for each trial.

After completing the 24th trial, participants were presented with a screen displaying a loading bar with a caption that read: “Accessing Database. Please Wait,” and after a few seconds, “Calculating Standardised NVR Score. Please Wait.” Participants were then presented with a screen providing performance feedback. The feedback information indicated the test population against which the participants’ score was compared (University Students), the average for the test population (100) and the participants own score; arbitrarily 77 in the failure feedback condition, or 122 in the success feedback condition. The screen also displayed a normal distribution curve indicating the percentages of scores for the test population falling 1–4 *SD*s above and below the mean. An arrow on the figure indicated the position of the participants’ score on the distribution, showing that it fell in either the bottom 16% (failure feedback) or top 16% (success feedback) of scores (see Figure 2).[Fig-anchor fig2]

After viewing their feedback, participants called the experimenter back into the room and were asked whether they needed assistance in interpreting their feedback. Regardless of participants response, the experimenter pointed out the participants score and the population mean, and informed them that their score was “very good (*not so good*), it puts you in the top (*bottom*) 16 odd percent of scores for the test population.” This was primarily intended to ensure that participants had understood the feedback, and was also expected to reinforce the impact of the manipulation by making the score public to the experimenter, and by ensuring that participants perceived their performance to be good or bad in relation to their peers.

###### Good/bad break manipulation

Participants were then asked to take part in an unexpected questionnaire study on “personality,” which involved “some questions regarding our experimental procedures and a short personality inventory.” The questionnaire also introduced the manipulation of a good versus bad break. Participants were verbally informed that “as the study was unexpected, and we may overrun the advertised time a little, we are going to give you the chance to win an extra £3 compensation.” Participants were then presented with a stack of 20 questionnaires and asked to pick one at random. Those in the good break (bad break) condition were informed that 30% (70%) of the questionnaires were winners. In actuality, as participants were pre-assigned to either a good or bad break, all of the questionnaires presented were either win or lose. This instruction was intended to potentially increase the impact of the breaks manipulation by indicating that the subsequent win or loss was against the odds. Participants then chose a questionnaire package, the top sheet of which included the PnR lottery procedure from Study 1a.

Participants then completed a questionnaire entitled “Experimental Feedback Form,” ostensibly so that we could “better understand participants’ experience of taking part in our research and improve upon procedure in future studies.” Before completing the items on the questionnaire, participants were asked to circle whether they won or lost the PnR lottery. Participants then answered the following six questions regarding the fairness/reasonableness of the PnR procedure: “How fair did you find the Peel n’ Reveal procedure used to decide whether you would receive extra compensation” (1 = *extremely unfair*, 9 = *extremely fair*); “How unreasonable to reasonable do you think the Peel n’ Reveal procedure is as a means of deciding whether participants will receive extra compensation” (1 = *extremely unreasonable*, 9 = *extremely reasonable*); “To what extent did you feel unfortunate to fortunate with the outcome of the Peel n’ Reveal Lottery” (1 = *very unfortunate*, 9 = *very fortunate*); “To what extent do you feel that you will receive reasonable compensation for your time in the lab today” (1 = *not at all*, 9 = *a great deal*); “To what extent did you feel dissatisfied to satisfied with the outcome of the Peel n’ Reveal Lottery” (1 = *very dissatisfied*, 9 = *very satisfied*) and “To what extent do you feel that the outcome of the Peel n’ Reveal Lottery was deserved” (1 = *not at all*, 9 = *a great deal*). These items were averaged to form a composite measure of how fair/reasonable participants perceived the PnR procedure. All six of these items loaded onto a single principal component (eigenvalue = 2.91, 48.45% of the variance accounted for; all component loadings > .55) and showed acceptable internal consistency (α = .79). An additional component was extracted from this analysis (eigenvalue = 1.32) but inspection of the loadings revealed no consistent or meaningful pattern among the items (i.e., a mix of positive and negative loadings). Finally, participants provided demographic information and the experimenter reentered the booth to inform them that the study was completed. Participants were probed for suspicion regarding the feedback and break manipulations and extensively debriefed. During the debriefing, participants in the failure feedback condition also completed an exercise in which they were required to write down several of their best qualities, which was intended to alleviate any lasting influence of the false feedback manipulation.

#### Study 2b

##### Participants

We recruited participants online (*N* = 190, 65% male, 0.5% unreported; *M*_age_ = 31.11 years, *SD*_age_ = 9.75) using MTurk.

##### Materials and procedure

Participants learned that the study concerned “personality, traits, and everyday experiences.” Representing our manipulation of self-esteem (cf. Wood et al., 2009), participants first completed a “content analysis survey” about either their “personal attributes” (low self-esteem condition) or “daily experiences” (control condition). Participants in the low self-esteem condition were asked to describe three things that they least liked about themselves and describe what the person who is closest to them would say are their worst two traits. Participants in the control condition were asked to describe three “everyday” features of their average day at work and what they wore yesterday and three days ago.

Next, participants completed the six-item DBOS used in Study 1b (αs > .89). Finally, to reduce any negative effects of recalling their worst traits, participants were asked to describe three of the successes and/or achievements in their life they were most proud of.

### Results and Discussion

Perceived fairness/reasonableness of the PnR procedure (Study 2a) was analyzed using a 2 (Test Performance Feedback: failure vs. success) × 2 (Experienced Break: good vs. bad) analysis of variance (ANOVA). These analyses revealed a main effect of breaks, *F*(1, 79) = 24.49, *p* < .001, η_p_^2^ = .234, such that participants who experienced a good break (won PnR) perceived the PnR procedure as more fair and reasonable. There was no significant main effect of feedback, *F*(1, 79) = 0.16, *p* = .69.

Shown in Figure 3, analyses revealed a Feedback × Breaks interaction, *F*(1, 79) = 6.02, *p* = .016, η_p_^2^ = .071.^2^ Follow-up analyses showed that the effect of experiencing a good or bad break on perceiving the PnR procedure as fair/reasonable was weaker among participants who received failure feedback, *t*(79) = 1.78, *p* = .08, than among participants who received success feedback, *t*(79) = 5.20, *p* < .001.^3^

In Study 2b, participants who described their worst traits and the things they least liked about themselves believed they were significantly more deserving of bad outcomes (*M* = 2.60, *SD* = 1.13) than participants in the control condition (*M* = 2.21, *SD* = 0.85), *t*(188) = 2.68, *p* = .008, *d* = 0.39.

**Figure 1 fig1:**
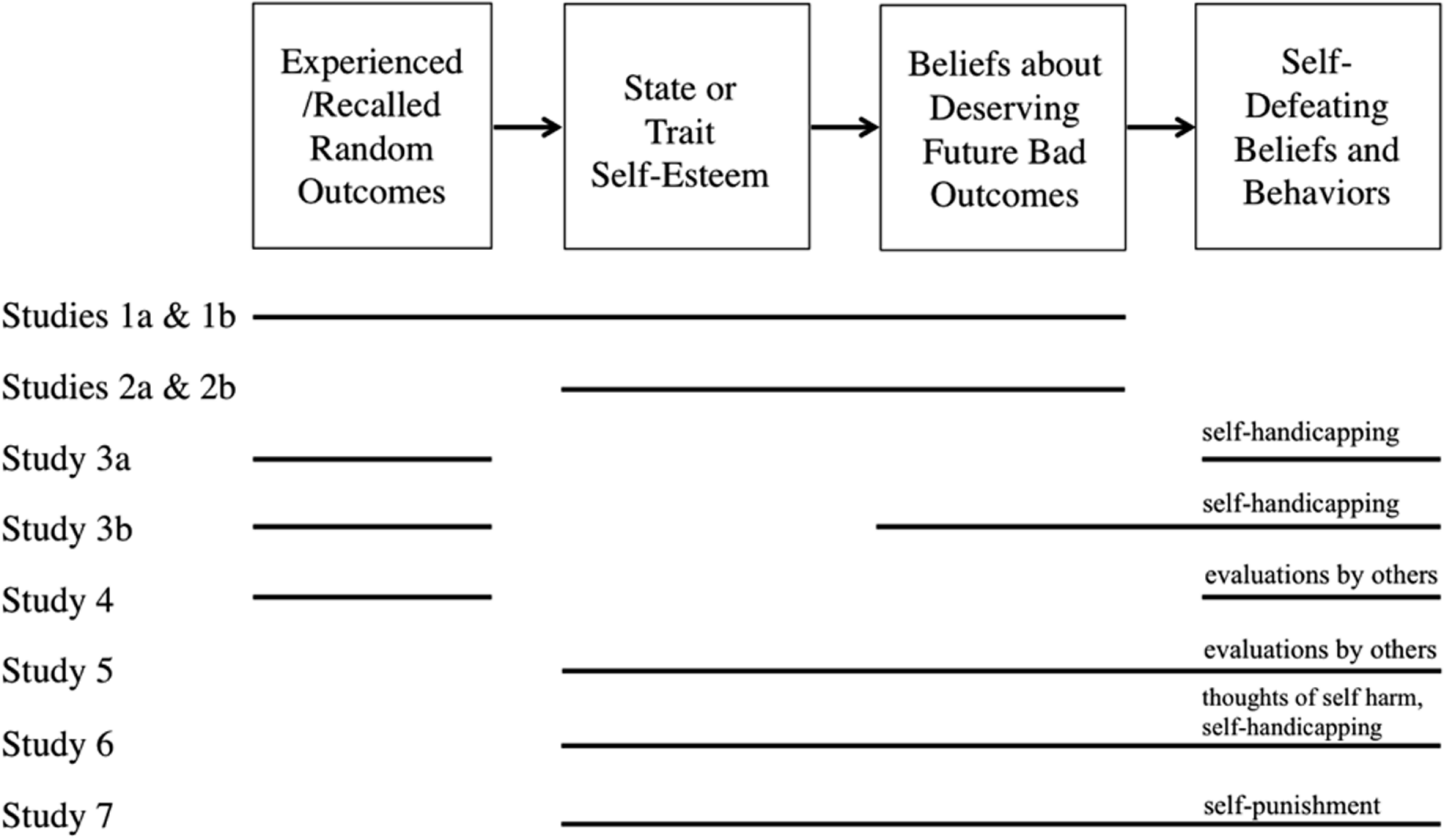
Conceptual framework for the studies. Solid lines show the variables that were measured or manipulated for each study. The specific self-defeating beliefs or behaviors measured in Studies 3a–7 are noted on the right-hand side of the figure.

**Figure 2 fig2:**
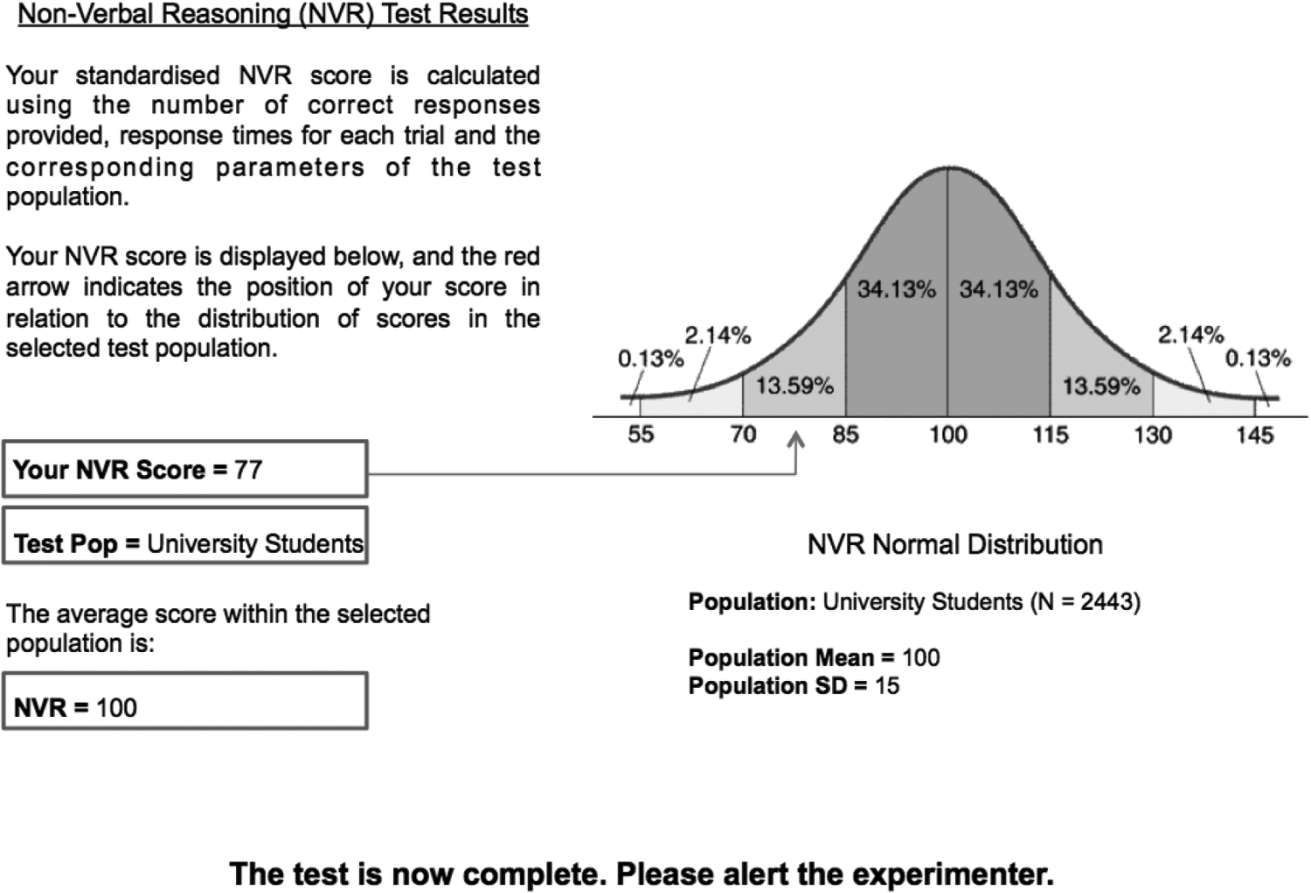
Feedback screen for the “failure” condition shown to participants at the end of the non-verbal reasoning (NVR) test in Study 2. Participants in the “success” condition received a NVR score of 120.

**Figure 3:**
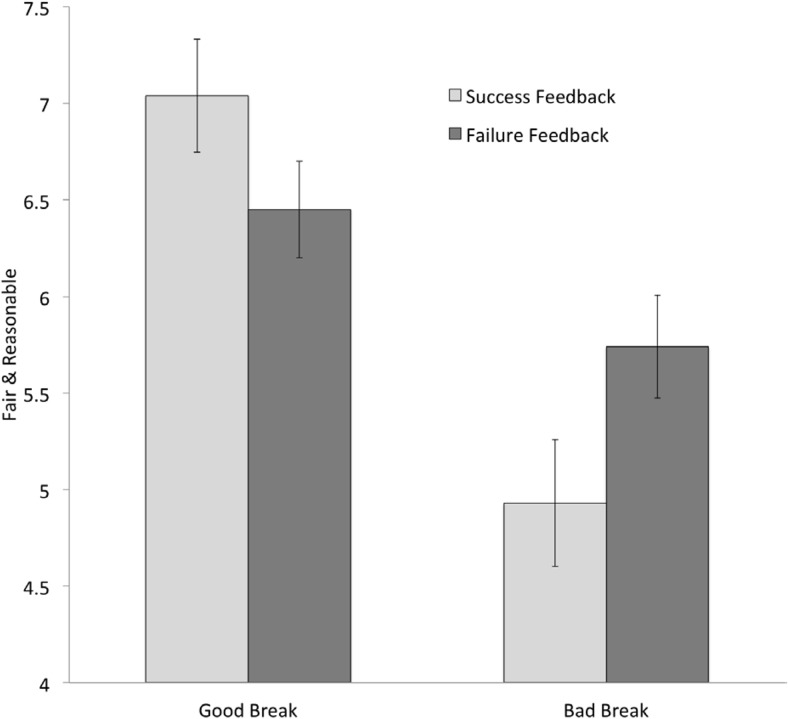
The effect of success versus failure feedback on the perceived fairness/reasonableness of a random break as a function of the valence of the experienced random break. Error bars show standard errors of the means.

Using different manipulations of self-esteem and different measures of deserving bad outcomes, Studies 2a and 2b provided converging experimental evidence for the causal role that self-esteem plays in people’s beliefs about deserving subsequent bad outcomes. Following our framework shown in Figure 1, we now turn our attention to examining the role that personal deservingness plays in the relations among self-esteem and self-handicapping (Studies 3 and 6), wanting others to evaluate the self negatively (Studies 4 and 5), thoughts of self-harm (Study 6), and self-punishment (Study 7).

## Studies 3A and 3B: Effects of Good/Bad Breaks on Self-Handicapping

In Studies 3a and 3b, we tested whether beliefs about deservingness brought about by experiencing/recalling bad breaks affects claimed self-handicapping. Self-handicapping involves claiming or creating an excuse or disadvantage prior to performing a task that may serve as a ready-made explanation in the event of failure (for reviews, see Higgins, Snyder, & Berglas, 1990; Hirt & McCrea, 2002; Rhodewalt, 2008). Self-handicapping is believed to serve a self-protective function because, in the short-term, it enables people to maintain positive self-esteem in light of task failure by attributing poor performance to a self-handicap instead of a lack of ability. Although self-handicapping has these short-term attributional benefits (Feick & Rhodewalt, 1997; McCrea & Hirt, 2001), it is ultimately self-defeating (Baumeister, 1997; McCrea & Flamm, 2012; Zuckerman, Kieffer, & Knee, 1998; Zuckerman & Tsai, 2005). As Baumeister (1997) noted, “what makes it qualify as a self-defeating behavior is that self-handicapping objectively increases the likelihood of failure” (p. 153). Moreover, Zuckerman and Tsai (2005) found that, over time, chronic self-handicappers were less well adjusted, reported an increase in substance abuse, and were less intrinsically motivated at work. Many studies have shown that lower self-esteem is related to increased self-handicapping (e.g., Finez, Berjot, Rosnet, Cleveland, & Tice, 2012; Rhodewalt, 1990; Spalding & Hardin, 1999; Zuckerman & Tsai, 2005). In Studies 3a and 3b, we tested the role that beliefs about deserving bad outcomes play in this link between self-esteem and self-handicapping. Specifically, using established experimental procedures (e.g., Hendrix & Hirt, 2009; McCrea & Hirt, 2011), we tested whether participants experiencing/recalling bad breaks (vs. good breaks) will engage in claimed self-handicapping (e.g., feeling stressed and tired) prior to taking a test purporting to assess their verbal intelligence (which is a meaningful and important attribute for most people; Tice, 1991).

### Study 3a

We first conducted an initial study to ascertain whether participants who experienced a bad (vs. good) break claim excuses for potential poor performance ahead of completing a standard non-verbal reasoning (NVR) test (Raven, 1938). Tice (1991) found that whether people low or high in self-esteem engaged in more or less behavioral self-handicapping ahead of an ability test (e.g., reducing practice) depended on whether the test was perceived as being able to diagnose only success or only failure. Finez et al. (2012), however, showed that this finding does not necessarily generalize to *claimed* self-handicapping, with people lower in self-esteem self-handicapping more regardless of whether a test of performance was pitched as being meaningful for only success or only failure. In Study 3a, then, participants completed the PnR lottery procedure we used in Study 1a and were told they would complete a NVR test. They were then given the opportunity to claim mitigating factors for potential poor performance on the test. Self-handicapping was operationalized as the extent to which participants self-reported mitigating circumstances for their potential poor performance on the NVR test.

### Study 3b

Study 3b was designed to extend Study 3a in two important ways: First, our theoretical perspective suggests that perceived deservingness of bad outcomes—in this case, deservingness of failing an ability test—should be one process that underlies the effect of the good/bad breaks on self-handicapping. In Study 3b, in addition to manipulating recalled bad/good breaks and measuring claimed self-handicapping, we assessed the degree to which participants believed they deserved to fail an impending NVR test. Second, because recalling bad (vs. good) breaks affects not only people’s self-evaluations but also their negative affect (cf. Studies 1b and 4), it is not clear if claiming excuses (e.g., feeling stressed and tired) ahead of an ability test represents self-handicapping per se or participants’ actual experiences of negative affect brought about by remembering their recent bad breaks. To address this potential issue, in Study 3b we crossed a manipulation of recalling bad/good breaks with a manipulation of whether participants learned that mitigating circumstances were known to negatively affect test performance or not (cf. Hendrix & Hirt, 2009; McCrea & Hirt, 2011). Claiming excuses, such as feeling tired and stressed, provides a valid handicap for poor performance when such mitigating circumstances matter, but not when they do not matter. Thus, if recalling bad breaks affects participants’ self-handicapping and not simply their reporting of negative affect, then we expected that recalling bad (vs. good) breaks would affect participants’ claimed excuses for potential poor performance only when they were told that mitigating circumstances were known to have detrimental effects on performance. Finally, we included pre-measured trait self-esteem for two reasons: (1) to ensure the reasoning test was perceived in such a way that participants lower in self-esteem engaged in more self-handicapping with the performance context we used (cf. Finez et al., 2012; Tice, 1991); and (2), consequently, as a covariate to take account of individual variation in the propensity for people low in self-esteem to report feeling stressed, tired, etc. under potentially threatening situations (see Spalding & Hardin, 1999).

### Method

#### Study 3a

##### Participants

We approached 62 people at various locations around the University of Essex to complete a “non-verbal reasoning” study. Two participants were not included in analyses because they completed the PnR lottery after the dependent measures. Another participant returned a blank questionnaire. The resulting sample consisted of 32 males and 27 females (*M*_age_ = 22.22 years, *SD*_age_ = 5.28). Participants were given a small candy bar and £3 for their participation.

##### Materials and procedure

Upon agreeing to participate for a small candy bar, we gave participants a questionnaire package, the cover sheet of which contained a consent form and the same PnR lottery procedure used in Study 1a. After giving their consent and winning or losing the PnR lottery, participants completed an easy, intermediate, and hard item taken from Raven’s (1938) Standard Progressive Matrices, ostensibly as practice for an upcoming “real” test of their intelligence. To facilitate the credibility of the cover story, the experimenter held additional questionnaires in view of the participant, ostensibly the upcoming test, on which were printed further matrices.

Following the practice items, participants circled whether they won or lost the PnR lottery. On the same sheet of paper was a “Mitigating Factors” questionnaire. Participants read:

Research shows that performance on standardized IQ tests, such as the one you are about to take, can be negatively influenced by a number of factors other than your actual ability (e.g., lack of sleep, stress). So that we can account for the presence of such mitigating factors in our analysis, please answer the following questions.

As our measure of claimed self-handicapping (cf. Hendrix & Hirt, 2009; Spalding & Hardin, 1999), participants then rated the extent to which they currently felt *tired*, *alert*, *well-rested*, *focused*, and *stressed* (1 = *not at all*, 7 = *very much*). Participants also indicated how many hours sleep they had the previous night (open-ended). Because the hours of sleep item was scaled differently from the remaining items, all items were standardized and averaged to form our measure of self-handicapping (higher values denote more self-handicapping; α = .74). Finally, participants were informed they would not actually be taking an intelligence test, debriefed, and paid £3 regardless of the outcome of the PnR lottery.

#### Study 3b

##### Participants

Participants were recruited online via Amazon’s MTurk (*N* = 367, 33% male, 0.5% unreported; *M*_age_ = 33.67 years, *SD*_age_ = 13.00).

##### Materials and procedure

Participants learned the study concerned “daily experiences, aspects of personality, and non-verbal reasoning ability, a component of general intelligence (or IQ).” Participants first completed Rosenberg’s SES (with the items rated on a 4-point scale ranging from 1 = *strongly disagree* to 4 = *strongly agree*) and, to facilitate the credibility of the cover story, the Ten-Item Personality Inventory (TIPI; Gosling, Rentfrow, & Swann, 2003). Next, participants learned about the upcoming non-verbal reasoning test, and completed the same three practice IQ test items from Study 3a.

Participants then completed the “daily experiences” part of the study, where they recalled either 4 good breaks or 4 bad breaks. To manipulate whether mitigating factors mattered or did not matter, participants then completed either an “additional factors questionnaire” or a “mitigating factors questionnaire.” Both questionnaires were the same except for the instructions given. In the “mitigating circumstances matter” condition, participants read the same instructions from Study 3a about how mitigating factors can affect test performance. In the “mitigating circumstances do not matter” condition, participants read:

Research shows that performance on standardized IQ tests, such as the one you are about to take, is not influenced by factors other than your actual ability. In keeping with convention, however, it is necessary for us to ask you about these factors, although they are very unlikely to influence your performance on the test. So that we can keep a record of these factors for comparison with other surveys, please answer the following questions:

Below these instructions were the same self-handicapping items used in Study 3a (which were standardized and averaged across the items; α = .81). Next, participants completed a questionnaire designed to assess their feelings about how deserving they felt of failing the upcoming IQ test (see Table 1; 1 = *strongly disagree*, 7 = *strongly agree*). Before completing the questionnaire, participants read: “We are also interested in people’s general beliefs and feelings about their anticipated performance on the non-verbal reasoning test. Please answer the following questions.” All seven items from this Deservingness of Failing Ability Test Scale loaded onto a single principal component (eigenvalue = 4.20, 59.85% of the variance accounted for; α = .89; see Table 1).

Finally, participants provided demographic information and, as a check on the mitigating circumstances matter manipulation, completed the item: “To what extent do you believe mitigating circumstances affect people’s test performances on ability tests?” (1 = *not at all*, 7 = *a great deal*).

### Results

#### Study 3a

An independent samples *t*-test showed that participants who experienced a bad break during the PnR self-handicapped to a greater extent (*M* = 0.20, *SD* = 0.63) than participants who experienced a good break (*M* = −0.21, *SD* = 0.64), *t*(57) = 2.44, *p* = .018, *d* = 0.64.

#### Study 3b

##### Manipulation check

A 2 (Breaks Recalled: good vs. bad) × 2 (Mitigating Circumstances: matter vs. do not matter) ANOVA on the manipulation check item confirmed that participants in the “mitigating circumstances matter” condition believed mitigating circumstances affect people’s test performances on ability tests to a greater extent (*M* = 5.28, *SD* = 1.36) than participants in the “mitigating circumstances do not matter” condition (*M* = 4.59, *SD* = 1.56), *F*(1, 361) = 20.00, *p* < .001, η_p_^2^ = .052. No other effects were significant (*p*s > .56).

##### Self-handicapping and perceived deservingness of failing test

Claimed self-handicapping was analyzed using a 2 (Breaks Recalled: good vs. bad) × 2 (Mitigating Circumstances: matter vs. do not matter) analysis of covariance (ANCOVA) with pre-measured self-esteem as a covariate. These analyses revealed one substantial outlier (studentized deleted residual = 4.66; Cook’s *D* that was 13.43 *SD* above the mean of Cook’s *D*) that was not included in this analysis. Including this data results in a weaker interaction effect for self-handicapping, *F*(1, 362) = 3.77, *p* = .053, η_p_^2^ = .01(see below).

**Figure 4 fig4:**
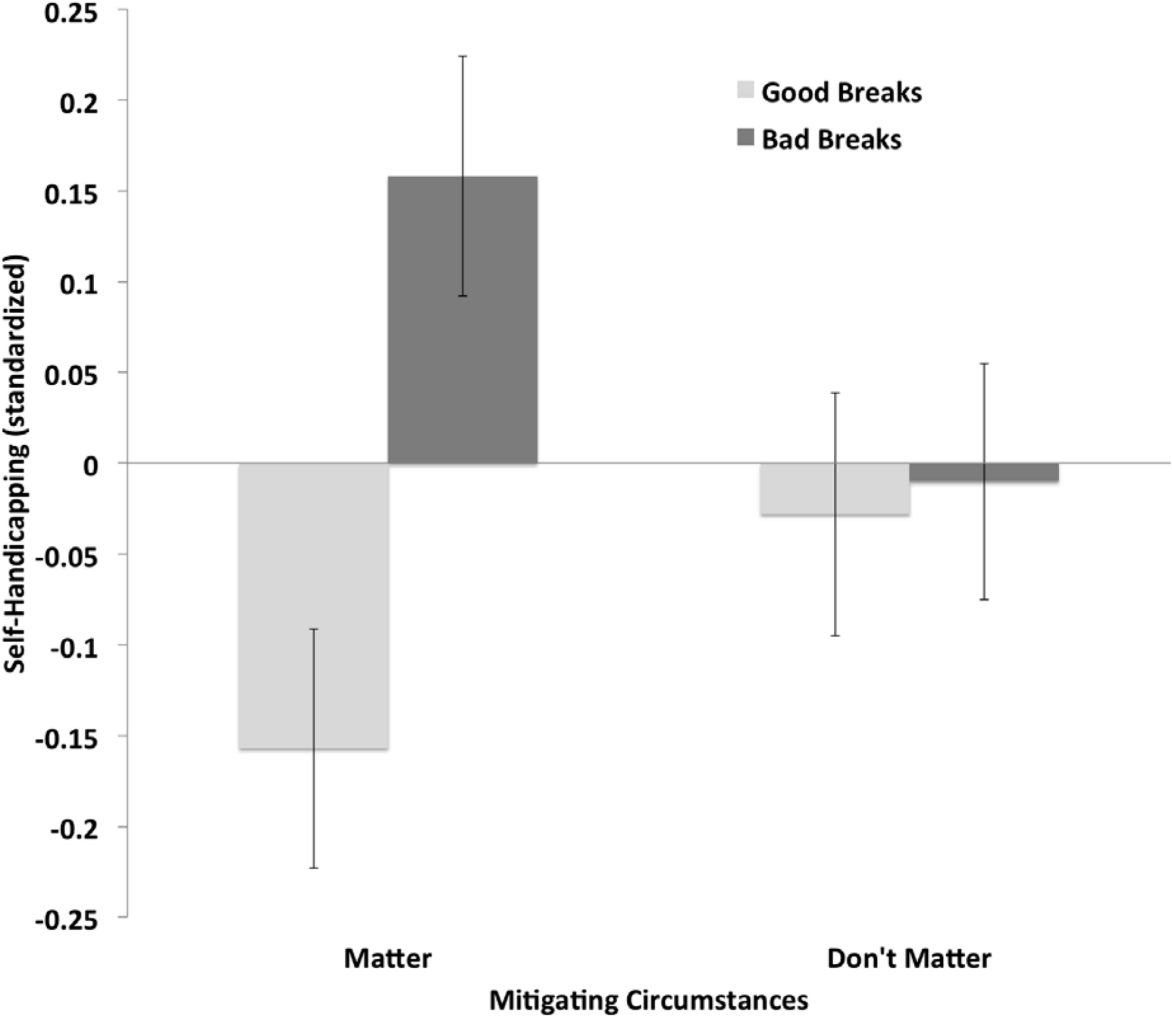
Effect of recalling bad versus good breaks on self-handicapping ahead of an intelligence test as a function of whether participants learned that mitigating circumstances affect test performance or not. Error bars show standard errors of the means.

The ANCOVA revealed a significant relation between self-esteem and participants’ self-handicapping, *F*(1, 361) = 78.11, *p* < .001, *r*_12_ = −.41, η_p_^2^ = .178, such that lower self-esteem related to higher ratings of claimed self-handicapping overall (cf. Spalding & Hardin, 1999). Analyses also revealed a significant Breaks Recalled × Mitigating Circumstances interaction for self-handicapping, *F*(1, 361) = 5.06, *p* = .025, η_p_^2^ = .014. Shown in Figure 4, participants who recalled bad (vs. good) breaks self-handicapped to a greater extent when mitigating circumstances mattered, *t*(361) = 3.38, *p* < .001, but not when mitigating circumstances did not matter, *t*(361) = 0.20, *p* = .80.[Fig-anchor fig4]

**Figure 5 fig5:**
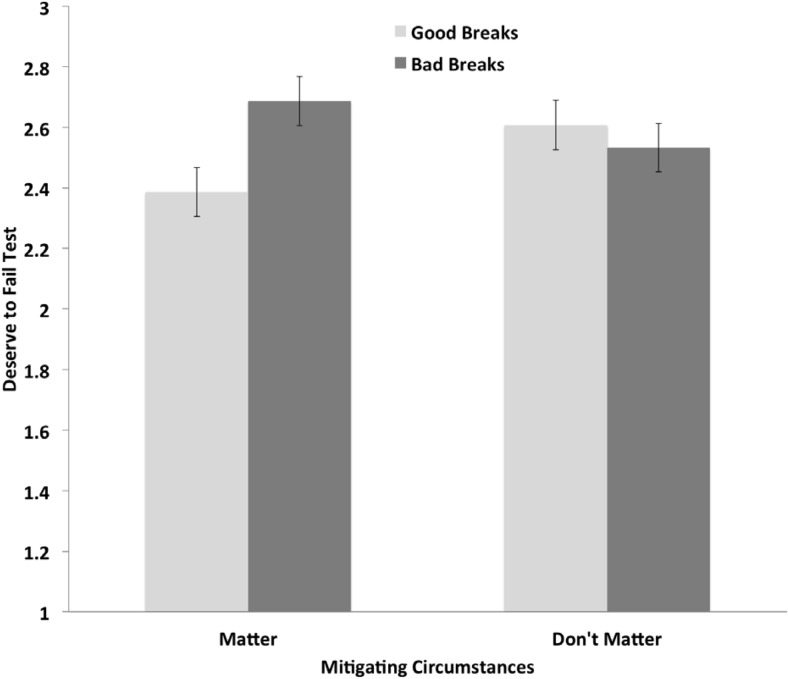
Effect of recalling bad versus good breaks on beliefs about deserving to fail the upcoming intelligence test as a function of whether participants learned that mitigating circumstances affect test performance or not. Error bars show standard errors of the means.

The same ANCOVA with beliefs about deserving to fail the upcoming test as the dependent variable revealed a significant effect of self-esteem, *F*(1, 362) = 260.01, *p* < .001, *r*_12_ = −.64, η_p_^2^ = .418, such that lower self-esteem related to greater beliefs about deserving to fail the test (cf. Studies 1a, 1b, and 4). Analyses also revealed a significant Breaks Recalled × Mitigating Circumstances interaction for beliefs about deserving to fail the test, *F*(1, 362) = 5.38, *p* = .021, η_p_^2^ = .015. Figure 5 shows that participants who recalled bad (vs. good) breaks believed they deserved to fail the test to a greater extent when mitigating circumstances mattered, *t*(361) = 2.63, *p* = .01, but not when mitigating circumstances did not matter, *t*(361) = −0.65, *p* = .51.[Fig-anchor fig5]

##### Moderated mediation analyses

Beliefs about deserving to fail the ability test correlated significantly with self-handicapping (*r* = .41, *p* < .001). Following Preacher, Rucker, and Hayes’s (2007) bootstrapping procedure for testing conditional indirect effects (Model 2), we explored whether deservingness beliefs mediated the effect of recalled good versus bad breaks on claimed self-handicapping depending on whether participants were told that mitigating circumstances mattered or did not matter. Using 10,000 resamples, analyses showed that beliefs about deserving to fail the test significantly mediated the effect of recalled breaks on self-handicapping when mitigating circumstances mattered (indirect effect = .04, 95% BCa CI [.01, .10]) but not when mitigating circumstances did not matter (indirect effect = −.01, 95% BCa CI [–.06, .02]).

The pattern of findings in Study 3 resonates with research showing that self-handicapping results from evaluative concerns about failure in situations known to increase such concerns (e.g., public self-focus, prevention focus; Hendrix & Hirt, 2009; Hirt, McCrea, & Kimble, 2000). We extend this work, however, by showing that (a) these concerns can reflect people’s perceived deservingness of failing when mitigating circumstances matter and (b) even recalling/experiencing random misfortunes is sufficient to trigger this process.

## Study 4: Recalling Good/Bad Breaks and Evaluations by Others

Studies 1a and 1b showed that participants who experienced/recalled bad (vs. good) breaks devalued their self-worth and believed they were more deserving of bad outcomes, and Study 3b showed that these processes contributed to claimed self-handicapping. Study 4 builds on these findings by examining another potential consequence of these changes in how people view their self-worth and deservingness: preferring that others appraise them negatively. A large body of research from self-verification theory (e.g., Swann, 2012; Swann, Rentfrow, & Guinn, 2002) has convincingly demonstrated that people are motivated to see that others view them as they view themselves, even if those self-views are negative. For example, Swann, Wenzlaff, Krull, and Pelham (1992) found that participants with negative self-views preferred that their dating partners and friends viewed them less favorably than participants with positive self-views. Although such strivings for self-verification are generally adaptive, in many instances they can be costly, such as when individuals with unrealistic negative self-views gravitate toward relationships (e.g., abusive partners) and situations (e.g., frustrating work environments; Wiesenfeld, Swann, Brockner, & Bartel, 2007) that further undermine their self-esteem (see Swann, 2012).

In Study 4, participants rated their specific beliefs about themselves, recalled either their recent good or bad breaks, and then rated how they wanted their close friends to view them. On the basis of the findings from Study 1b and the self-verification literature, we predicted that controlling for participants’ self-views, participants who recalled their recent bad breaks would prefer that their close friends viewed them less favorably than participants who recalled their good breaks.

### Method

#### Participants

Students from the University of Essex participated in a laboratory experiment for £3 or partial course credit (*N* = 85, 38.8% male; *M*_*age*_ = 20.56 years, *SD*_age_ = 2.90).

#### Materials and procedure

Participants were informed that the study concerned “personality and life events.” They first completed a Self-Attributes Questionnaire (SAQ; Pelham & Swann, 1989), which served as our pre-measure of trait self-esteem. For the SAQ, we asked participants to rate their attitudes about their activities and abilities relative to other university students their own age and gender using a 10-point scale ranging from *Bottom 5%* to *Upper 5%*. Participants provided self-ratings for 10 attributes/abilities: intellectual ability, social skills/social competence, artistic and/or musical ability, athletic ability, physical attractiveness, leadership ability, common sense, emotional stability, sense of humor, and discipline. The SAQ showed acceptable internal consistency (α = .73).

Next, participants completed a “life experiences questionnaire” where they recalled either 4 good breaks or 4 bad breaks (per Study 1b). Finally, they completed a “preferred appraisal by others” questionnaire that asked them to rate how they wanted their good friends to view them in terms of the same 10 attributes/abilities that they previously self-rated (α = .83) and a general appraisal by others item: “Ideally, I would like my good friends to view me:” (1 = *very negatively*, 8 = *very positively*). The specific appraisal and general appraisal scales correlated significantly (*r* = .29, *p* = .006) and were averaged to form one measure of appraisal by others scale (the general appraisals item was rescaled to a 10-point scale for this purpose). Higher values indicate greater preferences for close friends to view the self positively. Finally, participants completed the items from the PANAS (αs > .87) used in Study 1.

### Results

We ran an ANCOVA on participants’ preferred appraisals by their good friends with breaks recalled as a between-subjects factor and self-appraisals as a covariate. One participant did not complete the SAQ and was therefore not included in these analyses. Consistent with findings from the self-verification literature (Swann, 2012), there was a significant relation between participants’ self-appraisals and their preferred appraisals by others, *F*(1, 81) = 67.86, *p* < .001, *r*_12_ = .64, such that the less favorable participants viewed their own attributes/abilities overall, the less they preferred their close friends to view them favorably. Shown in Table 3, the manipulation of breaks recalled also exerted a significant effect: participants who recalled their bad breaks wanted their good friends to view them more negatively than participants who recalled their good breaks. This pattern of results was similar when the specific appraisals by friends and general appraisal by friends measures were analyzed separately.[Table-anchor tbl3]

ANCOVAs with self-appraisals as a covariate were performed to test the effect of recalling good or bad breaks on negative and positive affect. These analyses revealed a significant effect of recalled breaks on negative affect but not positive affect (see Table 3). The main effect of breaks on appraisals by close friends reported above held in a similar ANCOVA that also included positive and negative affect as covariates, *F*(1, 79) = 10.43, *p* = .002. This finding suggests that the effect of recalling good/bad breaks on participants’ preferred appraisal by their close friends is not specifically due to changes in general affect.

## Study 5: Self-Esteem, Deserving Bad Outcomes, and Evaluations by Others

Our previous studies showed consistent evidence that experiencing/recalling random and uncontrollable negative outcomes can change how people view their self-worth and beliefs about deserving bad outcomes, and these changes had demonstrable effects on how people wanted others to evaluate their personal worth and self-handicapping. Providing experimental evidence for the relation between self-esteem and beliefs about deserving bad outcomes, Study 2b showed that a manipulation of participants’ self-worth led them to deem a procedure that ultimately disadvantaged them as more fair/reasonable. In Study 4, we found that participants who recalled their bad (vs. good) breaks preferred that their close friends evaluated them more negatively. This finding indicates that not only can random breaks affect participants’ self-evaluations and perceived deservingness of bad outcomes (cf. Studies 1a and 1b), they can also influence corresponding judgments about how people want *others* to evaluate their attributes and qualities. In our final three studies, we augmented these experimental findings by capitalizing on existing individual variation in chronic self-esteem and beliefs about deserving bad outcomes to test the hypothesis that one of the reasons why self-esteem relates to self-defeating behaviors is because people lower in self-esteem generally believe they deserve bad outcomes (see Studies 2a and 2b).

In Study 5, we took an individual differences approach to zero in on deservingness as a process variable in the relation between low self-esteem and preferring that others view the self less favorably. We hypothesized that individual differences in self-esteem would positively, and beliefs about deserving bad outcomes would negatively, predict the extent to which participants wanted others to view them favorably. We also hypothesized that beliefs about deserving bad outcomes would feature in an indirect effect of self-esteem and the degree to which people wanted others to view them favorably.

### Method

#### Participants

Participants were recruited via Amazon’s MTurk to complete a survey about their personality and experiences (*N* = 142, 56% male; *M*_age_ = 32.73 years, *SD*_age_ = 11.64).

#### Materials and procedure

Participants first completed Rosenberg’s (1965) SES, using a scale ranging from 1 (*strongly disagree*) to 6 (*strongly agree*) for each item. Next, participants completed a nine-item scale we designed to measure participants’ general beliefs about deserving bad outcomes. The items were rated using a scale ranging from 1 (*strongly disagree*) to 6 (*strongly agree*). Shown in Table 1, this scale was adapted from the scale we developed for Study 1b, but the items were framed in general terms rather than how participants felt at the moment. A principal component analysis revealed only one substantive component explaining participants’ responses (eigenvalue = 4.41, 48.96% of the variance accounted for). These analyses were conducted on the collated data from Studies 5, 6, and 7 (these studies used the same deservingness of bad outcomes measure; total *N* = 461). An additional component was extracted from this analysis (eigenvalue = 1.32) but inspection of the loadings revealed no consistent or meaningful pattern among the items (i.e., a mix of positive and negative loadings).

Finally, participants completed a four-item measure assessing the degree to which they wanted others to view them favorably (see Swann, n.d.). These items were similar to the self-esteem items but were framed in terms of how the participants wanted *others* to view them (e.g., “I want others to have a positive attitude toward me”; “I want others to see that I am able to accomplish what I do”). The items were rated using a scale ranging from 1 (*strongly disagree*) to 6 (*strongly agree*).

### Results

**Figure 6 fig6:**
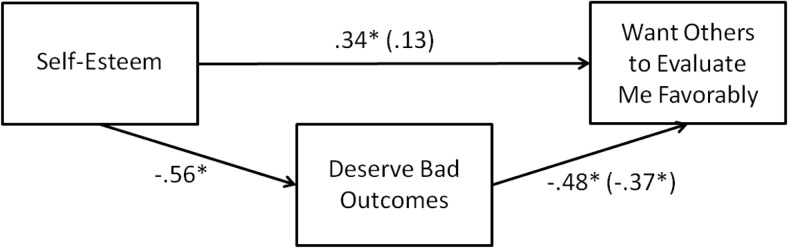
Mediational model predicting the preference for others to evaluate oneself favorably from beliefs about deserving bad outcomes and self-esteem (Study 5). Values show unstandardized path coefficients. * *p* < .05.

Shown in Table 4, correlation analyses showed that self-esteem, beliefs about deserving bad outcomes, and desired evaluations by others all correlated significantly with each other in the predicted directions. Multiple regression analyses regressing the desire for others to view one favorably onto self-esteem and beliefs about deserving bad outcomes revealed only deservingness as a significant predictor (see Figure 6). Bootstrapped mediation analyses (10,000 resamples) showed that beliefs about deserving bad outcomes was a significant mediator of the relation between self-esteem and wanting others to view the self favorably (indirect effect = .21, 95% BCa CI [.09, .34]). Adding to our Study 4 experimental findings, these findings indicate that a concern for deservingness is one mechanism that moves people from “I’m a bad person” to “I want others to view me negatively,” because, as we have argued and demonstrated experimentally (Studies 2a and 2b), people low in self-esteem feel more deserving of bad outcomes. Crucially, these results provided evidence that a concern with deservingness is one mechanism that links self-esteem to the desire for favorable evaluations by others.[Table-anchor tbl4][Fig-anchor fig6]

## Study 6: Self-Esteem, Deserving Bad Outcomes, Thoughts of Self-Harm, and Self-Handicapping

In Study 6, we focused on the mediating role that deservingness plays in the links between self-esteem and habitual self-handicapping and self-esteem and thoughts of self-harm. As mentioned, a large body of research has shown that people lower in self-esteem tend to engage in more habitual self-handicapping (e.g., Zuckerman et al., 1998). The habitual or chronic self-handicapper is someone who engages in a variety of behavioral and claimed excuses, such as withdrawing from achievement contexts, procrastination, and engaging in behaviors that are harmful in and of themselves (e.g., alcohol and drug consumption; see Zuckerman & Tsai, 2005). Our theoretical perspective suggests that one of the reasons why people low in self-esteem might self-handicap is because they believe they deserve bad outcomes. That is, we argue that people might engage in patterns of self-handicapping behavior to justify their beliefs that they deserve bad outcomes in life. If there is merit in this analysis, then we would expect individual differences in beliefs about deserving bad outcomes to statistically mediate the relation between self-esteem and chronic self-handicapping.

Physical self-harm has also been linked repeatedly to low self-esteem (e.g., Hawton, Rodham, Evans, & Weatherall, 2002; for a review, see Klonsky & Muelenkamp, 2007). But how does one move from believing “I’m a bad person” to “I want to harm myself”? Given our theoretical perspective and previous findings, we argue that deservingness might be one potent mechanism that allows people to move from self-derogation to thoughts of self-harming—that is, individuals who entertain thoughts of self-harm and engage in self-harming behaviors do so, in part, because they feel they deserve bad outcomes in life. This analysis fits well with research showing that self-punishment is one of the most prevalent reasons given by self-injurers for their self-injury (Klonsky, 2007; Nock, 2009). According to this self-punishment hypothesis, people engage in self-harm because it “provides a vehicle for punishing oneself for some perceived wrongdoing or responding to general self-hatred or self-deprecation” (Nock, 2010, p. 352). In support of this view, self-injury patients often report that one of the reasons why they self-injure is because they perceive themselves as bad and *deserving* of suffering (e.g., Adams, Rodham, & Gavin, 2005; Favazza, 1996; Walsh, 2006). This research, however, has been predominately descriptive and involving clinical samples. To our knowledge, little empirical work has examined the role that beliefs about deserving bad outcomes play in the links between low self-esteem and people’s thoughts of self-harm. Nevertheless, Nock (2010) states in his review of the clinical self-injury literature that achieving a better understanding of the psychological processes involved in these phenomena “represents an essential direction for future research” (p. 353).

To this end, along with chronic self-handicapping, we assessed participants’ self-esteem, beliefs about deserving bad outcomes in life, and how often they thought of harming themselves over the previous two weeks. We predicted that beliefs about deserving bad outcomes would mediate the relation between self-esteem and thoughts of self-harm and self-esteem and chronic self-handicapping. Because both chronic self-handicapping and self-harming have been found to correlate with depression (e.g., Hawton, Saunders, & O’Connor, 2012; Klonsky & Muelenkamp, 2007; Zuckerman & Tsai, 2005), we also included a measure of depressive symptomatology to explore the role that beliefs about deserving bad outcomes play in chronic self-handicapping and thoughts of self-harm over and above depression. That is, we tested whether beliefs about deserving bad outcomes uniquely predict thoughts of self-harm and chronic self-handicapping while controlling for depression.

### Method

#### Participants

Participants were recruited online via Amazon’s MTurk (*N* = 139, 29.5% male, 7.2% unreported; *M*_age_ = 30.05 years, *SD*_age_ = 11.56).

#### Materials and procedure

Participants were informed that they would complete a series of questionnaires that measured various aspects of their personality and how they felt about themselves. Participants completed the following measures in order:

##### Rosenberg’s SES

Participants completed the 10-item Rosenberg’s SES. The items were rated using a scale ranging from 1 (*strongly disagree*) to 4 (*strongly agree*).

##### Deservingness of Bad Outcomes Scale (DBOS)

Participants completed the nine-item DBOS we developed for Study 3b (see Table 1).

##### Patient Health Questionnaire–8 (PHQ-8)

The PHQ-8 is an eight-item measure designed to assess current depression in the general population (Kroenke et al., 2009). Participants were instructed to indicate how often over the last 2 weeks they were bothered by eight problems (e.g., “Feeling down, depressed, or hopeless”; “Trouble concentrating on things such as reading the newspaper or watching television”) using a scale ranging from 1 (*not at all*) to 4 (*nearly every day*). We used the average of the PHQ-8 items to assess individual differences in depressive symptomatology.

##### Thoughts of Self-Harm Scale

We constructed two items, which appeared at the end of the PHQ-8, to gauge participants’ thoughts of self-harm over the last 2 weeks (“Thoughts of harming, hurting, or injuring yourself” and “Wanting to experience physical pain”). As with the PHQ-8 items, these two items were rated using a scale ranging from 1 (*not at all*) to 4 (*nearly every day*). The two items were significantly correlated (*r* = .74, *p* < .001) and were averaged to form one measure of thoughts of self-harm.

##### Self-Handicapping Scale (SHS)

A disposition to self-handicap was measured using Rhodewalt’s (1990) 14-item SHS. The SHS assesses a variety of self-handicapping behaviors (e.g., “I suppose I feel ‘under the weather’ more often than most people”; “I tend to put things off until the last moment”), and has been shown to predict self-handicapping behaviors in a variety of contexts (Rhodewalt, 1990; Zuckerman et al., 1998). The items were rated on a scale ranging from 1 (*strongly disagree*) to 6 (*strongly agree*); responses were averaged to form one measure of a disposition to self-handicap.

Finally, after completing the measures participants were presented with a debriefing screen that included links to online resources if they felt they might be experiencing symptoms of depression (e.g., http://www.mentalhealthamerica.net/).

### Results

Shown in Table 4, correlation analyses showed that all of the measures correlated significantly with each other. Of note, both self-esteem and beliefs about deserving bad outcomes correlated significantly with thoughts of self-harm and self-handicapping.

Separate multiple regression analyses regressing thoughts of self-harm and self-handicapping onto the remaining measures revealed that only beliefs about deserving bad outcomes and depression uniquely predicted thoughts of self-harm and self-handicapping (see Table 5). These findings—that beliefs about deserving bad outcomes significantly predict thoughts of self-harm and self-handicapping over and above depression—are important because they suggest that rather than simply being a derivative or symptom of depression, deservingness beliefs uniquely contribute to these self-defeating thoughts and behaviors.[Table-anchor tbl5]

**Figure 7 fig7:**
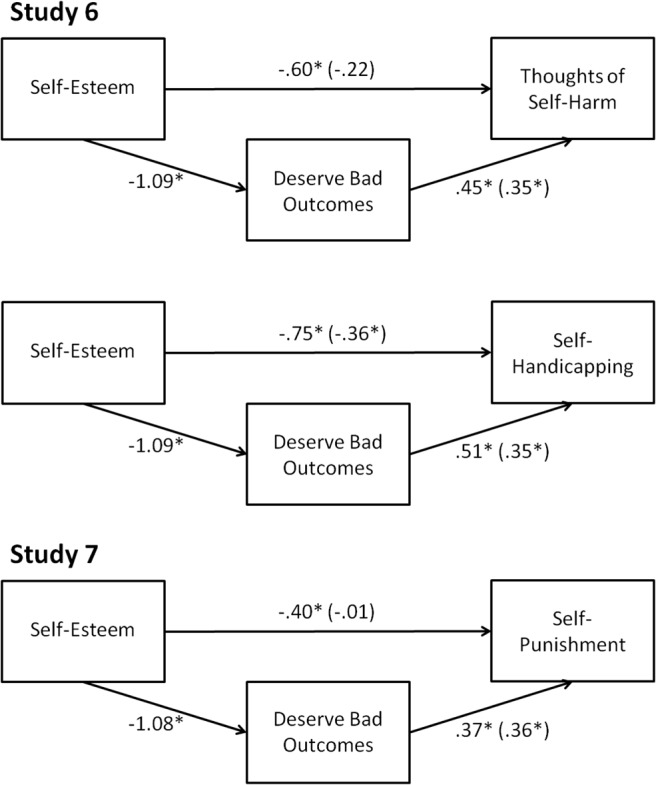
Mediational models predicting thoughts of self-harm and chronic self-handicapping (Study 6) and choosing to self-punish during an intelligence test (Study 7) from beliefs about deserving bad outcomes and self-esteem. Values show unstandardized path coefficients. * *p* < .05.

We conducted mediation analyses to examine whether beliefs about deserving bad outcomes mediated the links between self-esteem and thoughts of self-harm and self-esteem and self-handicapping. Shown in Figure 7, bootstrapping analyses with 10,000 resamples showed that beliefs about deserving bad outcomes significantly mediated the relations between self-esteem and thoughts of self-harm (indirect effect = −.38, 95% BCa CI [–.62, –.20]) and self-esteem and chronic self-handicapping (indirect effect = −.39, 95% BCa CI [–.60, –.21]). Further analyses showed that including depressive affect (PHQ-8) as a covariate in these bootstrapped mediation analyses did not change the significant indirect relations between self-esteem and thoughts of self-harm (indirect effect = −.22, 95% BCa CI [–.43, –.10]) and self-esteem and self-handicapping (indirect effect = −.26, 95% BCa CI [–.45, –.12]) through deserving bad outcomes. These findings show that beliefs about deserving bad outcomes mediate the relations between self-esteem and thoughts of self-harm and self-esteem and self-handicapping over and above the influence of depression.[Fig-anchor fig7]

## Study 7: Self-Esteem, Deserving Bad Outcomes, and Self-Punishment

Study 6 showed that one of the reasons why people entertain thoughts of self-harm is because they feel deserving of bad outcomes. In Study 7 we aimed to extend these findings by measuring actual self-punishment using a modified paradigm from the clinical literature (Roth, Rehm, & Rozensky, 1980; Rozensky, Rehm, Pry, & Roth, 1977). For example, Roth et al. (1980) found that mildly depressed participants were more willing to self-administer an aversive buzzer sound for believing they made incorrect responses during a memory test than participants lower in depression. We used a similar paradigm where after each question of an intelligence test, participants were given the choice to self-reward (receive positive feedback), self-punish (receive negative feedback), or receive no feedback if they believed they got the question correct or incorrect. As in our previous studies, we measured participants’ self-esteem and beliefs about deserving bad outcomes before they completed the intelligence test. We predicted that participants lower in self-esteem would choose to receive negative feedback more often, and, importantly, that beliefs about deserving bad outcomes would statistically underlie this relation.

### Method

#### Participants

Two samples of participants were recruited to partake in either a laboratory study or an online study. One sample consisted of 77 staff and students from the University of Essex, United Kingdom, who were recruited through a research participant database (55% females; *M*_age_ = 21.25 years, *SD*_age_ = 3.72). They were paid £3 for their participation. The other sample consisted of 103 participants recruited via MTurk to complete an online survey for a nominal payment (59% females, 1% unreported; *M*_age_ = 33.32 years, *SD*_age_ = 11.83).

#### Materials and procedure

Participants from both samples were told that the study involved a pilot test of a new short-form NVR test. Ostensibly as an unrelated study, participants completed a “personality” study where they completed Rosenberg’s (1965) SES and the nine-item DBOS from Study 6 (see Table 1). Participants from the laboratory sample completed these scales online at the point of registering for the study (i.e., prior to arriving to the laboratory), whereas participants in the online sample completed these measures immediately before completing the NVR test.

Participants then completed a computer-based NVR test, which involved solving a series of Raven’s (1938) matrices (see Studies 3a and 3b). For each matrix, participants were required to identify, from eight possible choices, the missing piece required to complete the pattern in accordance with the rule specified in the instructions. Participants in the online sample completed 12 matrices, whereas participants in the laboratory sample completed 20 matrices.

Participants first read instructions on how to complete the task and were shown an example matrix. On each trial, participants were first presented with a matrix set for a maximum of 20 s or until the advance button was pressed. A countdown timer was displayed on the screen. After providing their answer, participants were taken to an answer screen where the matrix set was no longer visible. They were then required to select the number (from 1 to 8) representing their chosen answer.

Participants were then taken to a further screen where they were given an opportunity to reward or punish themselves on the basis of whether *they* believed they had responded correctly to the matrix (no performance feedback was provided during the task). Specifically, participants were told:

If you believe your response was correct, please reward yourself by pressing the “Reward” button. If you believe your response was incorrect, please punish yourself by pressing the “Punish” button. If you are unsure if you gave the correct response, please press the “Unsure” button.

When “Reward” was selected, participants were shown a feedback screen for 1 s displaying the text “Well Done!” in large green font, and the next trial began. If “Punish” was selected, participants were shown “You’re Wrong!!” in large red font. Selecting “unsure” advanced to the next trial without displaying any feedback. The number of times participants opted to select the “punish” option served as our measure of self-punishment.^4^

### Results

Because participants across samples completed a different number of items during the NVR test (20 and 12), participants’ choices to self-punish and self-reward were standardized within samples prior to analyses. As might be expected, self-punishment was relatively uncommon overall, with participants choosing to self-punish on average 2.45 and 0.65 times in the laboratory and online samples, respectively (differences between samples here reflect the overall difficulty of the two NVR tests, with the laboratory sample including more difficult Raven’s matrices).

Preliminary moderated multiple regression analyses revealed that the correlations between self-esteem and self-punishment and deserving bad outcomes and self-punishment did not differ significantly between the samples (*p*s > .72), so the data were collated and analyzed together. Shown in Table 4, correlation analyses showed that both self-esteem and beliefs about deserving bad outcomes correlated significantly with the number of times participants chose to self-punish, such that lower self-esteem and higher beliefs about deserving bad outcomes related to more self-punishment. A multiple regression analysis regressing self-punishment onto self-esteem and deserving bad outcomes as predictors revealed one substantial outlier who was removed from analyses (studentized deleted residual = 5.16; Cook’s *D* that was 9.95 *SD* above the mean of Cook’s *D*). Including these data reveal similar correlations between self-esteem and self-punishment (*r* = −.19, *p* < .01) and deserving bad outcomes and self-punishment (*r* = .25, *p* < .01) as those reported in Table 4.

Interestingly, shown in Table 4, beliefs about deserving bad outcomes correlated negatively with test performance (i.e., percentage correct). The correlations with self-reward were weaker overall (cf. Roth et al., 1980), with only deserving bad outcomes correlating significantly with self-reward.

Bootstrapped mediation analyses (10,000 resamples) showed that beliefs about deserving bad outcomes statistically mediated the relation between self-esteem and self-punishment (see Figure 7; indirect effect = −.39, 95% BCa CI [–.70, –.15]). This pattern remained largely unchanged when test performance was included as a covariate (indirect effect = −.34, 95% BCa CI [–.62, –.11]). Moreover, multiple regression analyses with self-esteem and percentage correct on the test as predictors of self-punishment showed that the *c* path (self-esteem to self-punishment) remained significant while controlling for percentage correct on the test (*B* = .37, β = −.19, *SE* = .14, *p* = .009). Multiple regression analyses showed that the *a* path (self-esteem to deserving bad outcomes) also remained significant while controlling for test performance (*B* = −1.05, β = −.68, *SE* = .08, *p* < .001). The finding that this mediation pattern holds while controlling for test performance suggests that participants higher in beliefs about deserving bad outcomes were engaging in self-punishment and not simply concluding that they got the questions wrong. Importantly, these findings extend our Study 6 results by showing that concerns about deservingness play a role in how people move from the perception of lower personal worth to actually choosing to receive self-punishment.

## General Discussion

A long history of social psychological research has convincingly demonstrated that people are motivated to view their world as orderly and predictable (Kay et al., 2008)—a world where both they and others get what they deserve and deserve what they get (Lerner, 1980). The present work tested the idea that people who are lower in self-esteem might engage in self-defeating behaviors because they feel deserving of bad outcomes, even if those outcomes are brought about by chance and happenstance.

Findings from seven studies provided evidence for this idea. Participants who experienced/recalled random bad (vs. good) breaks devalued their state self-worth, which, in turn, increased their beliefs about deserving bad outcomes (Studies 1a and 1b).

Across our studies we found consistent evidence that self-esteem and beliefs about deservingness are highly related—bad (good) people deserve bad (good) outcomes. Study 2a offered experimental evidence for this link between self-esteem and personal deservingness by showing that a manipulation of self-worth led participants to deem a completely random and uncontrollable bad (good) break as more (less) fair and reasonable. That is, in spite of how seemingly irrational such judgments might be, participants reported a *random and uncontrollable* bad or good break as more or less fair if they received feedback that they failed or succeeded an intelligence test. Of course, from a rational perspective, such notions as “fairness” and “deserving” ought not to come into play when considering one’s chance outcomes—after all, such events are by definition unforeseeable and uncontrollable—but in light of the functional significance of the motive to view one’s outcomes as deserved rather than random, the fact that people might be moved to deem their bad breaks as deserved as a function of their self-worth becomes less puzzling. Study 2b extended this finding in a different way by showing that recalling one’s least favorable attributes (vs. control) increased the extent to which participants believed they deserved bad outcomes in life.

These changes in participants’ state self-worth and perceived deservingness had consequences for how participants (a) wanted others to evaluate their personal worth and attributes (Study 4), and (b) their self-handicapping behavior ahead of an ability test (3a and 3b). In Study 4, participants who recalled their bad (vs. good) breaks preferred that others appraised them less positively, and Study 5 offered important mediational evidence that this process of moving from “I’m a bad person” to “I want others to view me negatively” is guided, in part, by participants’ feeling that they deserve bad outcomes.

Study 3b showed that when mitigating circumstances mattered, participants who experienced/recalled bad breaks claimed excuses for potential failure during an ability test to a greater extent than participants who experienced/recalled good breaks, and these effects were linked to participants self-evaluative concerns about deserving to fail. In Study 6, we found that the same processes operated when considering the link between trait self-esteem and *chronic* self-handicapping: participants lower in self-esteem reported chronic excuse-making and patterns of behavioral self-handicapping partly because they felt deserving of bad outcomes in life.

In our final two studies we investigated participants’ thoughts of physical self-harm (Study 6) and self-punishing behavior (Study 7). In Study 6, beliefs about deserving bad outcomes mediated the relation between self-esteem and thoughts of self-harm, and this pattern held even when controlling for depression (which is a major precursor to self-injurious thoughts and behaviors; Klonsky & Muelenkamp, 2007). In Study 7, when given the choice to self-reward, self-punish, or do nothing during an intelligence test, participants lower in self-esteem opted to give themselves negative feedback more often than participants higher in self-esteem. As in our previous studies, beliefs about deserving bad outcomes statistically mediated this relation between self-esteem and self-defeating behavior.

### Trade-Offs Between Deservingness and Self-Defeat

Across our studies we found that people adopted a variety of beliefs and behaviors that are ultimately self-costly and self-defeating. Indeed, wanting others to evaluate the self negatively may lead some people to tolerate abusive partners (Swann, 2012), self-handicapping leads to maladjustment over time (Zuckerman & Tsai, 2005), and thoughts of self-harm can lead to actual direct injuring of the body (Nock, 2010). As we noted at the start of our article, these behaviors are at odds with the rationalistic view that humans are primarily motivated by self-preservation and the pursuit of self-interest. Why, then, would people be willing to adopt such self-defeating beliefs and behaviors? We put forward and empirically tested the idea that people who adopt these beliefs and engage in these behaviors do so, in part, because they are motivated to sustain the sense that people—including oneself—get what they deserve. Thus, our conceptual analysis and empirical findings resonate with Baumeister and Scher’s (1988; see also Baumeister, 1997) analysis that rather than being driven by a motivation for primary self-destruction, people who engage in self-defeating behaviors do so as a trade-off. In the case of the current findings, the trade-off is between the adaptive and functional belief that one’s world is fair, predictable, and orderly—a belief that provides the enabling psychological context for people to pursue their long-term goals with confidence (Callan, Harvey, & Sutton, 2014; Callan, Shead, & Olson, 2009; Hafer, 2000; Laurin et al., 2011)—and engaging in behaviors that are actually costly to the self to sustain that belief. That is, the motivation to see that people get what they deserve can lead to patterns of behavior that are ultimately self-costly, even though they are the result of an adaptive process that can be triggered by even random and uncontrollable negative life events.

### Limitations and Future Directions

One of the strengths of our work is that we adopted a diverse set of dependent measures and methods to triangulate on our hypotheses, but this broad, multi-method approach came at the expense of more focused investigations of the various self-defeating behavior patterns we tested. Thus, we have only scratched the surface of the various ways concerns about deservingness may play a role in wanting others to evaluate the self less favorably, self-handicapping, and self-harm over time and circumstance. In Studies 4 and 5, we found that people preferred others to evaluate them less favorably when they recalled their recent bad breaks and believed they deserved bad outcomes. What has yet to be investigated, however, is the extent to which these processes can contribute to actual negative feedback seeking by people with more negative self-views (e.g., actively choosing to receive negative over positive feedback from close others; Swann, Hixon, & De La Ronde, 1992). Such work may lead to an increased understanding of the processes involved self-verification, including how the perceived accuracy of social feedback might moderate the links between self-esteem, perceived deservingness of bad outcomes, and actual feedback seeking (see Bosson & Swann, 1999).

In Study 6 we found that concerns about deservingness was an important predictor of self-handicapping. Using a longitudinal design, Zuckerman and Tsai (2005) found that low self-esteem related to more self-handicapping over time, but that the use of self-handicapping also engendered a loss of self-esteem over time. On the basis of our theoretical analysis and current findings, we speculate that beliefs about deserving bad outcomes and self-handicapping might also reinforce each other over time, such that believing one deserves bad outcomes as a function of low self-esteem can lead to patterns of self-handicapping behavior that further diminishes one’s self-esteem and deservingness. Such longitudinal investigations might also speak further to the direction of influence between self-esteem, deserving bad outcomes, and self-defeat to supplement the current correlational and experimental findings.

For the obvious ethical reasons, in Study 6 we examined participants’ retrospective reports of their thoughts of self-harm rather than their actual self-injurious behaviors. Of course, it is important to investigate the reasons why people have recently thought about physically harming themselves (see Nock, 2010), but recent methodological advancements may allow future research to test the role that deservingness plays in *real time* self-injurious thoughts and behaviors (e.g., ecological momentary assessments with smartphones; see Nock, Prinstein, & Sterba, 2009). Such investigations may lead to further evidence for the self-punishment hypothesis of self-harm in addition to what we provided in the context of retrospective thoughts of physical self-harm.

Our results are consistent with our theoretical analysis in that participants, at least in the immediate context, engaged in self-defeat following their experiences of random negative experiences, and we provided evidence that one of the reasons they did so was because of their concerns about deservingness. What our findings do not speak to, however, are the accumulative, longer-term consequences of bad breaks and other negative events on people’s self-esteem, beliefs about deservingness, and patterns of self-defeating behaviors. By and large, most of our participants did not adopt self-defeating beliefs and engage in self-defeating behaviors in absolute terms, which reflects that fact that a large majority of people evaluate themselves favorably (Diener & Diener, 1995). That is, even though we showed changes in people’s self-esteem as a function of their random and uncontrollable bad breaks, participants generally did not feel deserving of bad outcomes or engage in self-defeat, because their self-esteem was still within the positive range in absolute terms. In fact, it was generally only our participants who were very low in self-esteem who, for example, entertained thoughts of physical self-harm (Study 6) and opted to punish themselves for potentially poor performance (Study 7).

What, then, might lead someone to hold especially high beliefs about deserving bad outcomes in life? One possibility is that although an experience of a single random bad event may influence people’s beliefs about deserving bad outcomes in the shorter-term as we have shown, it is the “wear and tear” of constantly experiencing bad breaks and other random and uncontrollable negative life events on people’s self-evaluations and emotional well-being (Charles, Piazza, Mogle, Sliwinski, & Almeida, 2013) that lead people into more chronic beliefs about deserving bad outcomes in life. Although we did not investigate these longer-term consequences, we offered a theoretical perspective that speaks to their importance, and we designed measures of beliefs about deserving bad outcomes that could be used in future investigations of these processes.

Finally, from a deservingness perspective, the belief that one deserves bad outcomes requires a less favorable view of the self. Thus, we suspect that the effects we report here—that is, lower self-esteem affects self-defeat through beliefs about deserving bad outcomes—are likely limited to those self-defeating behaviors that occur because of lower self-esteem or within contexts that are self-evaluative or self-relevant (cf. Wood et al., 2009).^5^ Along with identifying esteem-threat and emotional distress, Baumeister (1997) highlighted how self-regulation failures can lead to self-defeating behaviors. To the extent that people engage in some forms of self-defeat because of, for example, a loss of impulse control may have more to do with self-regulatory breakdown than their concerns about deservingness. Nonetheless, our findings complement Baumeister’s analysis by suggesting that perceived deservingness of future bad outcomes, even in the wake of mundane and uncontrollable bad breaks, is an important mechanism underlying the link between low self-esteem and self-defeat.

### Theoretical and Practical Implications

Researchers interested in the psychology of justice and deservingness have highlighted how the motive to view one’s world as stable and orderly can impact how they evaluate others’ outcomes and deservingness, such as reasoning backward from a victim’s random ill-fate to infer her personal worth (Lerner & Simmons, 1966) to perceiving “bad” people as deserving their random negative experiences (Callan et al., 2006). Much less research, however, has focused on whether the same processes operate in the contexts of one’s own random experiences and deservingness. If this motive is essential for people’s long-term goal pursuits, then one might expect to find that people’s reactions to their own fates as deserved might parallel their reactions to the fates of others as deserved. In Lerner’s (1977) early conceptualizations of the justice motive, deservingness for others and deservingness for the self are intimately intertwined, as pursing one’s long-term goals with a measure of confidence requires the belief that one’s social and physical environment is such that outcomes are distributed to those who deserve them rather than being governed by randomness. Across seven studies, we provided empirical support for the idea that in at least some contexts, a concern for deservingness for the self affects people’s reactions to their own outcomes in much the same way as they respond to the outcomes of others.

It is also interesting to consider how these processes may manifest at the group level. System justification theory (Jost, Banaji, & Nosek, 2004) has noted many examples of low-status groups attributing their disadvantaged status to inferior abilities and other characteristics of the ingroup, rather than pure discrimination or misfortune. Because these examples of so-called ingroup derogation are difficult to explain via prevalent models of social identity, these phenomena have garnered considerable interest from researchers. Some theorists explain ingroup derogation as the result of powerful groups convincing the less powerful to buy into legitimizing myths that justify extant power differences (Sidanius & Pratto, 1999)—that is, they explain it as resulting from the motivations and goals of high-status group members and their efforts to preserve their power and advantage. Another possibility, however, and one that is entirely consistent with theoretical treatments of system justification theory (Jost et al., 2004), is that cases of ingroup derogation may also be due to motivations on the part of the disadvantaged group members themselves—namely, their motivations to preserve general beliefs in deservingness. The research reported here, although limited to individual-level phenomena, speaks to the feasibility of this latter account.

Besides their theoretical importance, our findings point to possible intervention and treatment strategies for people seeking help for their self-defeating beliefs and behaviors (e.g., self-injurers). That is, the current findings offer empirical support for the potential importance of considering an individual’s beliefs about deserving bad outcomes as one source of his or her patterns of self-defeating behavior. Of course, more research is needed on these practical applications of the current findings, but helping clients understand how their concerns about personal deservingness might influence their self-harming behavior may prove to complement existing treatment strategies.

## Figures and Tables

**Table 1 tbl1:** Scale Items, Principal Component Loadings, and Communalities for the Beliefs About Deserving Bad Outcomes Scales

Scale items	Component loading	Communality
6-item State Deservingness of Bad Outcomes Scale (Studies 1b and 2b)		
1. Right now, I’d like to feel better about myself than I usually do, but deep down, I don’t feel I deserve to.	.75	.56
2. I feel unworthy of succeeding right now.	.87	.75
3. Right now, I feel I deserve to fail in life.	.88	.77
4. Right now, I feel that I deserve all of the good things life has to offer.^a^	.74	.55
5. Right now, I feel I deserve to do poorly in life.	.86	.75
6. Right now, I feel I deserve to do well in life.^a^	.81	.65
Deservingness of Failing Ability Test Scale (Study 3b)		
1. I feel that I deserve to do well on the ability test.^a^	.68	.46
2. I feel that I deserve to do poorly on the ability test.	.78	.62
3. Right now, I feel deserving of all the good things life has to offer.^a^	.71	.51
4. I feel I have a good shot at achieving a good score on the ability test.^a^	.84	.70
5. I feel confident about my ability to perform well on the ability test.^a^	.82	.67
6. I feel that I deserve to fail this ability test.	.81	.65
7. Right now, I do not feel deserving of positive outcomes.	.76	.57
9-item Deservingness of Bad Outcomes Scale (Studies 5, 6, and 7 collated)		
1. I feel that I deserve all of the good things life has to offer.^a^	.64	.60
2. I feel that I deserve to do poorly in life.	.73	.58
3. I often feel unworthy of my successes.	.76	.57
4. I often feel deserving of my failures.	.73	.66
5. When I suffer a setback, I sometimes think that I had it coming to me.	.71	.73
6. I feel that I deserve to do well in life.^a^	.70	.76
7. I often feel that I deserve the good breaks that happen to me.^a^	.70	.70
8. I often feel that I deserve the bad breaks that happen to me.	.69	.63
9. I’d like to feel better about myself than I usually do, but deep down, I don’t feel I deserve to.	.64	.51
^a^ Reverse scored.		

**Table 2 tbl2:** Means and Standard Deviations for the Measures Employed in Studies 1a and 1b by Breaks Conditions and Intercorrelations Among the Measures

Measures	Breaks manipulation				Intercorrelations			
	Bad breaks	Good breaks	*t*	*d*	1	2	3	4
Study 1a: Experienced breaks								
1. Self-esteem	4.68 (0.61)	5.01 (0.61)	2.35*	0.56	—			
2. Deserve bad outcomes	2.16 (0.80)	1.80 (0.72)	2.06*	0.49	−.70**	—		
3. Negative affect	1.58 (0.64)	1.66 (0.67)	0.56	0.13	−.17	.03	—	
4. Positive affect	3.39 (0.78)	3.60 (0.75)	1.20	0.28	.38**	−.40**	−.46**	—
Study 1b: Recalled breaks								
1. Self-esteem	4.32 (1.24)	4.78 (0.91)	3.18**	0.43	[.94]			
2. Deserve bad outcomes	2.41 (1.15)	2.00 (.88)	2.93**	0.40	−.84**	[.91]		
3. Negative affect	1.69 (0.79)	1.42 (0.67)	2.76**	0.38	−.61**	.52**	[.92]	
4. Positive affect	2.82 (1.07)	3.10 (1.08)	1.91	0.26	.64**	−.51**	−.41**	[.93]
*Note.* Higher values indicate more of each construct (e.g., higher self-esteem). Standard deviations are presented in parentheses. Alpha reliabilities are presented in brackets.								
* *p* < .05. ** *p* < .01.								

**Table 3 tbl3:** Adjusted Means for the Measures Employed in Study 4 by Breaks Condition and Intercorrelations Among the Measures

Measures	Recalled breaks manipulation				Intercorrelations			
	Bad breaks	Good breaks	*F*	η_p_^2^	1	2	3	4
1. Self-appraisals (premeasured)					—			
2. Preferred appraisals by others	7.87 (0.09)	8.30 (0.092)	10.69**	.12	.64**	—		
3. Negative affect	1.72 (0.088)	1.39 (0.090)	7.02**	.08	−.38**	−.33**	—	
4. Positive affect	3.00 (0.125)	3.14 (0.128)	0.65	.01	.33**	.11	−.14	—
*Note.* Higher values indicate more of each construct. Standard errors of the adjusted means are presented in parentheses.								
** *p* < .01.								

**Table 4 tbl4:** Descriptive Statistics and Intercorrelations for Measures Employed in Studies 5, 6, and 7

Measures	*M* (*SD*)	1	2	3	4	5
Study 5						
1. Self-esteem	4.47 (1.00)	(.94)				
2. Deserve bad outcomes	2.56 (0.81)	−.70**	(.86)			
3. Favorable evaluations by others	5.01 (0.80)	.42**	−.49**	(.93)		
Study 6						
1. Self-esteem	3.09 (0.59)	(.89)				
2. Deserve bad outcomes	2.47 (0.94)	−.69**	(.88)			
3. PHQ-8	1.97 (0.69)	−.62**	.55**	(.85)		
4. Thoughts of self-harm	1.40 (0.79)	−.46**	.53**	.64**	(.85)	
5. SHS	3.13 (0.82)	−.54**	.58**	.56**	.39**	(.85)
Study 7						
1. Self-esteem	3.12 (0.50)	(.85)				
2. Deserve bad outcomes	2.39 (0.78)	−.69**	(.85)			
3. Self-reward	0 (1.00)	.11	−.17*	—		
4. Self-punish	0 (1.00)	−.21**	.30**	−.43**	—	
5. % correct on IQ test	59 (21)	.11	−.20**	.28**	−.17*	—
*Note.* Higher values indicate more of each construct. Self-esteem was assessed on a 6-point scale in Study 5 and on a 4-point scale in Studies 6 and 7. Where applicable, alpha reliabilities are presented in parentheses along the diagonal. PHQ-8 = Patient Health Questionnaire–8; SHS = Self-Handicapping Scale.						
* *p* < .05. ** *p* < .01.						

**Table 5 tbl5:** Summary of Multiple Regression Analyses Predicting Thoughts of Self-Harm and Self-Handicapping (Study 6)

Predictor variables	Thoughts of self-harm			Self-handicapping		
	*b*	*SE*	β	*b*	*SE*	β
1. Self-esteem	.10	.13	.07	−.16	.14	−.12
2. Deserve bad outcomes	.27	.08	.32**	.31	.09	.35**
3. PHQ-8	.62	.10	.54**	.40	.12	.34**
4. Self-handicapping	−.07	.08	−.07			
5. Thoughts of self-harm				−.07	.09	−.07
Overall statistics	*F*(4, 130) = 27.35, *p* < .01, *R*^2^ = .46			*F*(4, 130) = 24.85, *p* < .01, *R*^2^ = .43		
*Note.* PHQ-8 = Patient Health Questionnaire–8.						
** *p* < .01.						

## Footnotes

1 A principal component analysis of the three deservingness items revealed that the last item (“I feel I deserve good luck”) did not load with the other two items (loading = .24 vs. .76 and .82). The item also did not significantly correlate with either the average of the other two deservingness items (*r* = .04, *p* = .73) or the SES (*r* = .18, *p* = .13) and was therefore dropped from the Deservingness of Bad Outcomes Scale. This scale was extended and improved in our subsequent studies (see Table 1).

2 Although our analysis strategy was to perform these analyses on the composite of the six items we measured, one reviewer wondered whether the pattern we report here is similar for those items more specific to fairness/deservingness. The same analyses using only the average of the “fairness” and “deservingness” items (*r* = .41, *p* < .001) revealed the same Feedback × Breaks interaction pattern, *F*(1, 79) = 5.72, *p* = .019, η_p_^2^ = .068.

3 Another way to articulate this interaction pattern is to transform the Feedback × Breaks interaction into a main effect of the feedback manipulation by reverse scoring the fair/reasonable scores within the good breaks condition only and conducting the same 2 × 2 ANOVA as above (see Abelson, 1995). Looking at the pattern of findings in this way, participants who received failure feedback rated the bad/good break as more/less fair (*M* = 4.64, *SD* = 1.61) than participants who received success feedback (*M* = 3.87, *SD* = 1.70), *F*(1, 79) = 6.02, *p* = .016, η_p_^2^ = .071. There was no significant interaction in these analyses, *F*(1, 79) = 0.16, *p* = .69, indicating that the absolute effect of success versus failure on perceived fairness was not significantly different between the good and bad breaks conditions.

4 Participants in the laboratory sample also learned that they would be completing a “mental dexterity” experiment after the NVR test during which they would either construct a toy from an educational science kit or write a short essay about the experience of making toys from such kits. Whether participants learned they would construct the toys or write a short essay did not significantly affect participants’ choices to self-punish or self-reward during the NVR test (*p*s > .70).

5 Indeed, two initial studies unexpectedly revealed that self-esteem did not correlate significantly with how much participants were willing to self-administer electrical stimulations (*r* = .04, *p* = .77; *N* = 54) or taste hot sauce (β = −.002, *p* = .49, controlling for liking of hot sauce and expectations of hotness; *N* = 60). Thus, in light of the current findings, it is important to consider patterns of self-defeating behaviors that are relevant to participants’ self-views for experiences of misfortune or deservingness to serve as contributing factors.
